# Imine Crosslinked, Injectable, and Self‐Healing Fucoidan Hydrogel with Immunomodulatory Properties

**DOI:** 10.1002/adhm.202405260

**Published:** 2025-04-18

**Authors:** Asma Talib Qureshi, Shajia Afrin, Saad Asim, Muhammad Rizwan

**Affiliations:** ^1^ Department of Biomedical Engineering University of Texas Southwestern Medical Center Dallas TX 75235 USA; ^2^ Department of Biomedical Engineering Michigan Technological University Houghton MI 49931 USA; ^3^ Department of Ophthalmology University of Texas Southwestern Medical Center Dallas TX 75235 USA

**Keywords:** fucoidan, hydrogel, injectable, immunomodulation, self‐healing

## Abstract

Biomaterials with inherent anti‐inflammatory properties and the ability to foster a pro‐regenerative environment hold significant promise for enhancing cell transplantation and tissue regeneration. Fucoidan, a sulfated polysaccharide with well‐documented immune‐regulatory and antioxidant capabilities, offers strong potential for creating such biomaterials. Yet, there is a lack of engineered fucoidan hydrogels that are injectable and provide tunable physicochemical properties. In this study, the ability of fucoidan to undergo periodate‐mediated oxidation is leveraged to introduce aldehydes into backbone (oxidized fucoidan, OFu), enabling the formation of reversible, imine‐crosslinks with amine‐containing molecules such as gelatin. The imine‐crosslinked OFu‐gelatin hydrogel provided excellent control over gelation rate and mechanical properties. Counter‐intuitively, OFu‐gelatin hydrogel exhibited excellent long‐term stability (≥28 days), even though imine crosslinks are known to be relatively less stable. Moreover, the OFu‐gelatin hydrogels are self‐healing, injectable, and biocompatible, supporting cell culture and encapsulation. Furthermore, fucoidan hydrogels displayed immune‐modulatory properties both in vitro and in vivo. This innovative injectable fucoidan hydrogel presents a versatile platform for applications in tissue engineering and regenerative medicine.

## Graphical abstract

This study develops an injectable fucoidan‐based hydrogel with tunable properties for tissue engineering. By oxidizing fucoidan (OFu) to introduce aldehydes, imine crosslinks are formed with amine‐containing polymers, enabling self‐healing, injectability, and long‐term stability (≥28 days). The OFu‐gelatin hydrogel demonstrated biocompatibility, immune‐modulatory effects, and controlled gelation, offering a promising platform for regenerative medicine and cell transplantation.

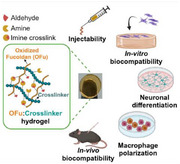

## 1 Introduction

Fucoidan, an FDA‐approved food supplement, is a natural sulfated polysaccharide derived from various species of brown seaweed.^[^ ^1^ ^]^ It has received significant interest in various biomedical applications, including tissue engineering, due to its remarkable biocompatibility, anti‐inflammatory, and immunomodulatory properties.^[^ ^2^, ^3^, ^4^ ^]^ Fucoidan has an innate potential to interact with cells of the innate and adaptive immune systems, as evidenced by its interaction with macrophages through Toll‐like receptors, dendritic cells, and T‐lymphocytes, enabling it to modulate immune responses.^[^ ^5^, ^6^, ^7^ ^]^ It exerts anti‐inflammatory effects by downregulating the expression of nuclear factor kappa B (NF‐κB), a key mediator of inflammation, and has been reported to inhibit pro‐inflammatory cytokines.^[^ ^8^, ^9^ ^]^ Due to these properties, fucoidan can mitigate inflammatory responses, thereby making it an ideal candidate to develop an immunomodulatory biomaterial such as injectable hydrogels. These hydrogels can potentially enhance tissue regeneration by delivering biotherapeutics while also modulating the local microenvironment to create a more pro‐regenerative niche. However, injectable fucoidan hydrogels optimized for tissue engineering applications such as transplantation of highly susceptible cells are still lacking, highlighting the need for dedicated efforts to bridge this gap.

Hydrogels are composed of hydrophilic polymers and are a key class of biomaterials for tissue engineering due to their ability to mimic natural tissues and their tunability to deliver biotherapeutics, such as bioactive molecules and cells. Fucoidan has been mainly used as an additive in various other hydrogels to improve hydrogel function. For instance, fucoidan has been reported to be incorporated into a hydrogel‐based delivery system comprised of chitosan, collagen, and β‐glycerophosphate for sustained release at the injury site.^[^ ^10^ ^]^ Tailoring the fucoidan content in a hydrogel composed of chitosan, fucoidan, and PEG di‐benzaldehyde improved the mechanical properties, viscoelasticity, and hydrogel stability, enabling a sustained release of polyphenols from the network.^[^ ^11^ ^]^ Fucoidan has also been leveraged to load growth factors (VEGF) and chemokines (SDF‐1) in chitosan/fucoidan‐based nanoparticles that were subsequently incorporated into a composite of chitosan, gelatin, and sodium β‐glycerophosphate to develop a temperature responsive injectable hydrogel for in situ vascularization.^[^ ^12^ ^]^ However, the lack of chemical interaction of fucoidan molecules with the hydrogel matrix is likely to result in the diffusing out of fucoidan from hydrogel, leading to lower stability and long‐term hydrogel functionality.^[^ ^10^, ^13^ ^]^ Moreover, only a few studies have focused on the design and development of injectable fucoidan‐based hydrogels to maximally leverage its immune‐modulatory and anti‐oxidant properties.^[^ ^14^, ^15^ ^]^ Therefore, to bridge this gap, we aimed to develop an injectable, highly stable fucoidan hydrogel for tissue engineering and regenerative medicine applications.

Several types of crosslinking chemistries have been utilized to obtain the injectable hydrogels with desirable mechanical and functional properties. Reversible covalent crosslinking has gained significant prominence due to its ability to form dynamic crosslinks in biomaterial networks. Unlike permanent covalent crosslinking, it offers the formation of reversible bonds that introduce shear thinning and injectable behavior in the hydrogel.^[^ ^16^, ^17^, ^18^, ^19^, ^20^ ^]^ For example, imine crosslinks formed by the reaction between an aldehyde and an amine group are stable at physiological pH. However, these bonds can also undergo degradation under certain conditions, allowing the release of encapsulated drugs and reforming, permitting the material to self‐heal without the influence of any external factor.^[^ ^21^, ^22^, ^23^ ^]^ This is a crucial property for injectable biomaterials, as it allows the material to undergo gelation postextrusion, thereby retaining the structural integrity of the hydrogel. Imine crosslinked polysaccharide‐based hydrogels have also been developed to impart immunomodulatory properties. However, this requires the addition of adjuvants such as metal ions or immunomodulatory peptides.^[^ ^24^, ^25^ ^]^ Imine‐crosslinked hydrogels can also bind with tissues due to the ability of aldehydes to react with amine groups present in tissues. Due to these properties, imine crosslinking is a powerful tool for developing injectable biomaterials for tissue engineering applications.^[^ ^22^, ^23^ ^]^

**Figure 1:**
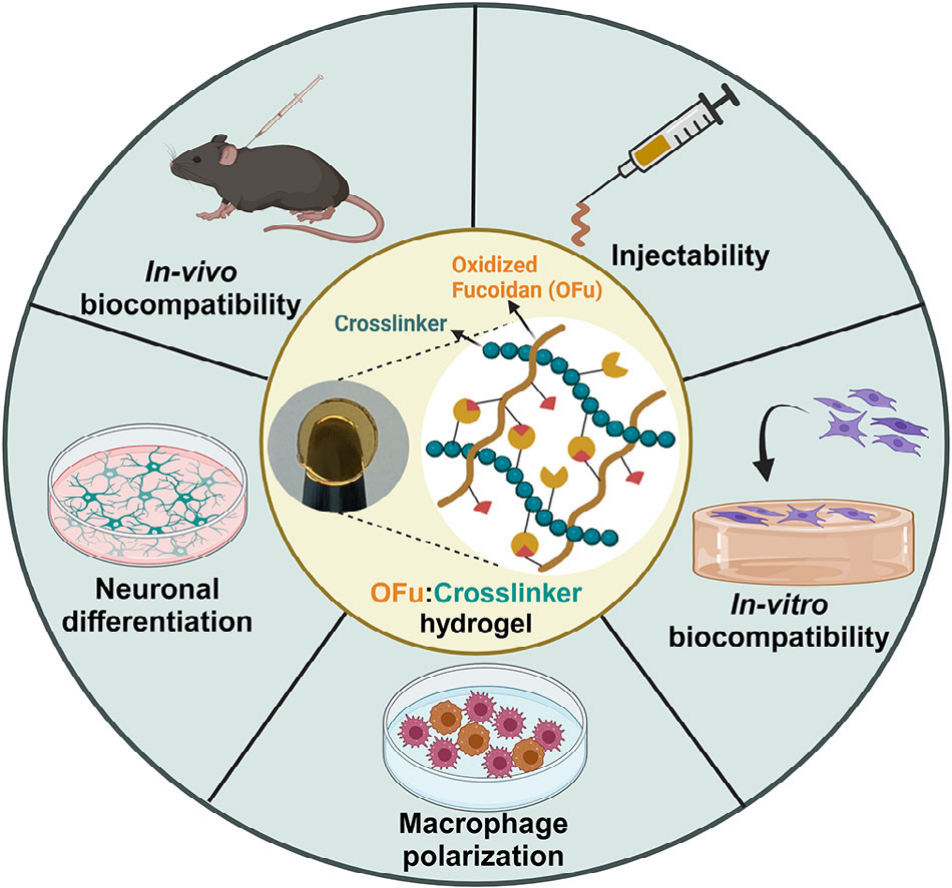
Schematic representation of imine crosslinked fucoidan hydrogel and its potential for biomedical applications.

To leverage the ability of the fucoidan to provide anti‐inflammatory signals, in this study, we developed injectable fucoidan hydrogels and demonstrated its tunability, injectability, and innate immune‐modulatory potential (**Figure** **1**). We chemically modified fucoidan to introduce aldehyde groups in its structure through periodate oxidation. These aldehyde groups were crosslinked with amine‐containing polymers, leading to the formation of fucoidan hydrogels with reversible dynamic imine crosslinks. These imine crosslinks impart self‐healing property to OFu hydrogels making them an ideal candidate for minimally invasive cell transplantation applications to central nervous system (CNS). As a pilot study, we tested the biocompatibility of these OFu‐based hydrogels with PC‐12 cells that serve as a model cell line for neuronal differentiation, in both 2D and 3D environments. Fucoidan in its native state is a brown opaque substance, with limited light transmission through it. This is a major limitation while analyzing the cell behaviors such as cell growth, spreading, migration, and in vivo real‐time imaging. Therefore, we improved the optical clarity of our hydrogels to visualize and monitor the cells cultured therein. The mechanical and degradation profiles of fucoidan hydrogels were tuned, and the hydrogels were characterized for injectability and in vitro biocompatibility. Moreover, CNS injuries are characterized by a hostile inflammatory microenvironment that disrupts the cellular homeostasis, limits regeneration after injury, and cause progressive neuronal loss.^[^ ^26^, ^27^, ^28^ ^]^ Fucoidan is known to provide a neuroprotective role as it has an innate potential to regulate the mitochondrial oxidative phosphorylation and offer protective effects against neuroinflammation, mitochondrial dysfunction and microglial activation.^[^ ^29^, ^30^ ^]^ Therefore, in this study, we also assessed the ability of the fucoidan in injectable hydrogel form to provide anti‐inflammatory environment using both in vitro and in vivo experiments. Together our results position injectable fucoidan hydrogels as a novel anti‐inflammatory biomaterial for potential neuro‐regeneration repair.

## 2 Results and Discussion

### 2.1 Imine Crosslinked Fucoidan Hydrogels Possess Tunable Gelation Rate and Mechanical Properties

**Figure 2:**
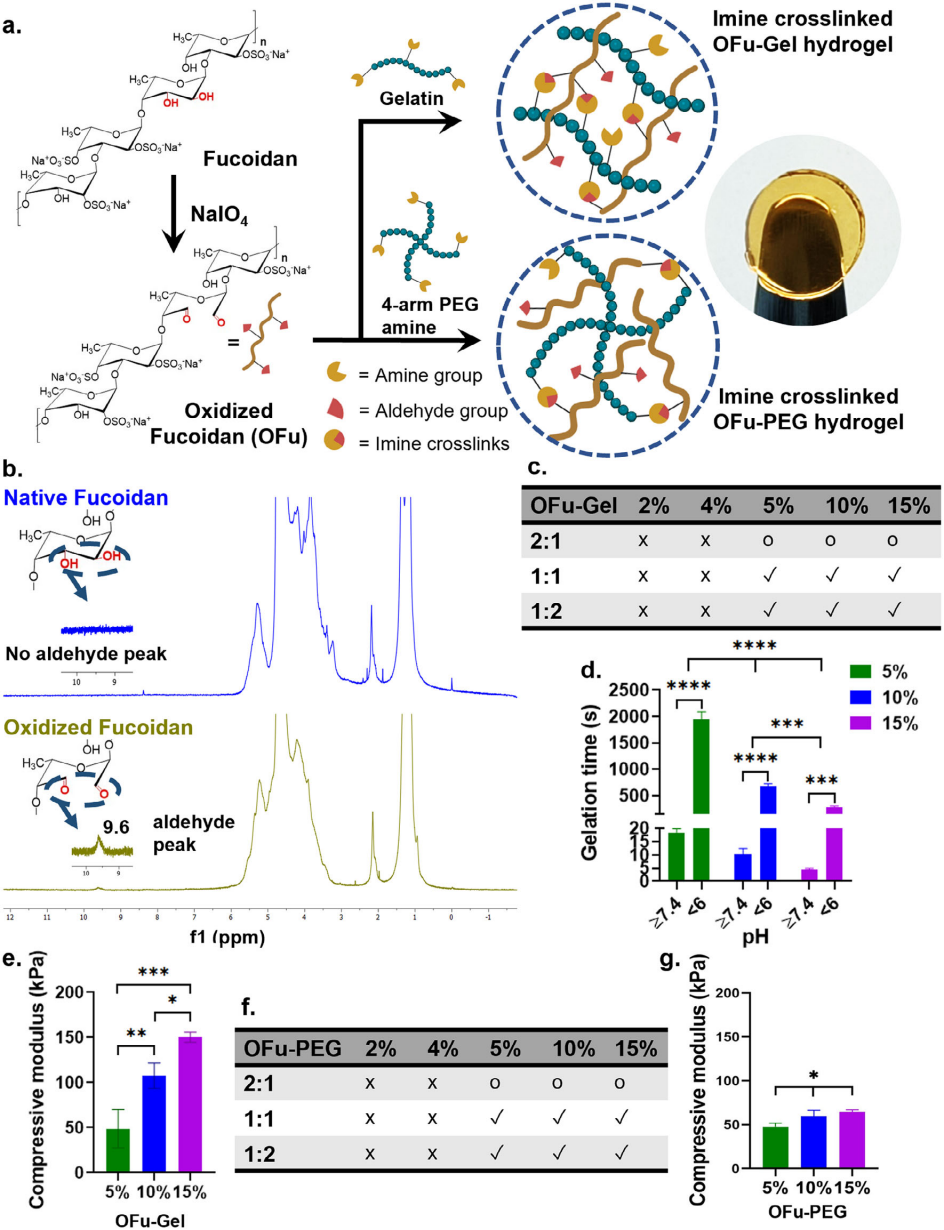
(a) Schematic illustration of chemical modification of fucoidan using sodium periodate that introduced two aldehyde groups at C2 and C3 positions of 1→4 linked fucose monomer. The aldehydes on OFu can further react with amine groups present on gelatin or 4‐arm PEG amine to form imine (‐C = N‐) crosslinked OFu‐Gel or OFu‐PEG hydrogels, respectively. (b) 1H‐NMR of native and oxidized fucoidan. (c) Effect of prepolymer concentrations of OFu and Gel on gelation of 2%, 4%, 5%, 10%, and 15% OFu‐Gel hydrogels. (x = no gelation; o = partial gelation; ✓ = complete gelation). (d) The effect of pH (pH < 6 and ≥7.4) and hydrogel concentration (5%, 10% and 15%) on the gelation time of OFu‐Gel hydrogels, p**** < 0.0001, p*** < 0.001, two‐way ANOVA, Sidak's multiple comparisons test. (e) Compressive strength of 5%, 10%, and 15% OFu‐Gel hydrogels. (f) Effect of prepolymer concentrations of OFu and PEG on gelation of 2%, 4%, 5%, 10%, and 15% OFu‐PEG hydrogels. (x = no gelation; o = partial gelation; ✓ = complete gelation). (g) Compressive strength of 5%, 10%, and 15% OFu‐PEG hydrogels. p*** < 0.001, p** < 0.01, p* < 0.05, one‐way ANOVA, Tukey's multiple comparisons test. *n* = 3, mean ± SD for all.

We utilized the hydroxyl groups in the fucoidan to introduce aldehydes via oxidation using sodium periodate – a widely used oxidizing agent for polysaccharides.^[^ ^31^, ^32^ ^]^ The reaction between fucoidan and sodium periodate resulted in bond cleavage between C2 and C3 of (1→4)‐linked six‐membered fucose ring allowing the oxidation of hydroxyl groups into aldehydes forming oxidized fucoidan (OFu) (**Figure** **2**a). Subsequently, the OFu was mixed with amine‐containing gelatin (Gel) biopolymer or 4‐arm Polyetylene Glycol (PEG) amine to form imine‐crosslinked OFu‐Gel or OFu‐PEG hydrogels, respectively (Figure 2a).^[^ ^31^ ^]^ To determine the degree of fucoidan oxidation, we used hydroxylamine hydrochloride titration method, which indicated an average of ≈33 ± 0.49% oxidation of fucoidan after 8 h of reaction, monitored from three independent oxidation reactions (Table S1, Supporting Information). The degree of oxidation can be tuned by varying different parameters, such as molar ratio of sodium periodate to polysaccharide or temperature, or by changing the reactions time.^[^ ^33^, ^34^ ^]^ Furthermore, the modification of fucoidan was validated by 1H‐NMR of native and oxidized fucoidan. The appearance of an aldehyde peak at 9.6 ppm in oxidized fucoidan, which was initially missing in native fucoidan, indicated the oxidation of fucoidan (Figure 2b). It is worth pointing out that the reaction of sodium periodate with polysaccharides is known to reduce the molecular weight of polysaccharides.^[^ ^35^, ^36^, ^37^ ^]^ Our oxidized fucoidan yield was ≈50% after dialysis using ≈10–12 kDa cut‐off tubes, suggesting that the oxidation process reduces the molecular weight, resulting in smaller fucoidan chains that are removed during dialysis, thereby lowering the yield. These findings align with previous reports. However, the quantitative reduction in the average molecular weight of fucoidan following oxidation requires further investigation.

To analyze the gelation, we first investigated the use of gelatin as a natural biomolecule crosslinker that contains amine groups. The molecular weight distribution of both fucoidan and gelatin used in this study are reported to be between 50 and 100 kDa.^[^ ^38^, ^39^, ^40^, ^41^ ^]^ The gelation parameters such as OFu to gelatin ratio and the pH of the prepolymer solutions were varied. The OFu to gelatin (w/v %) ratio was varied from 2:1 to 1:1 and 1:2, and the gelation was observed qualitatively by visual inspection and by pipetting the prepolymer solutions. Notably, when OFu concentration was kept higher than the gelatin concentration, i.e., 2:1, it led to incomplete gelation. However, mixing the prepolymer solutions in equal ratios or with high proportion of gelatin led to complete gelation of OFu and gelatin (Figure 2c). This is in line with the previous findings where an amine deficit led to a lower gel stability of hyperbranched polyglycerol (hPG‐NH_2_) and polyethyleneglycol dialdehyde (PEG‐DA) hydrogels and the unreacted PEG‐DA molecules remained in the solution state.^[^ ^42^ ^]^ Furthermore, we also noted that the lowest concentration of OFu and gelatin to form a hydrogel was 5% as further reducing the concentrations (e.g., 2% and 4%) did not form a hydrogel (Figure 2c). Presumably, the prepolymer chains of oxidized fucoidan and gelatin in a diluted prepolymer solution had the functional moieties too distant that they failed to establish a crosslinked network of polymer chains leading to no hydrogel formation.

The polymer content and the pH of the polymer solution plays a major role in imine crosslinking, as it regulates the formation and stability of these dynamic covalent crosslinks.^[^ ^22^, ^43^ ^]^ To tune the gelation rate of OFu‐Gel hydrogels, we varied the concentration of the prepolymer solution from 5% to 15%, and the pH of the prepolymer solutions from slightly acidic (≤6) to physiological pH of 7.4. We noted a significant decline in the gelation time as the prepolymer concentrations were increased from 5% to 15% (Figure 2d). Furthermore, the gelation for OFu‐Gel hydrogels was significantly delayed in an acidic environment compared to physiological pH (Figure 2d). This is evident from the gelation time of OFu‐Gel hydrogels that dropped significantly from ≈1940 s to ≈18 s for 5%, ≈683 s to ≈10 s for 10%, and ≈268 s to ≈3 s for 15% OFu‐Gel hydrogels by changing the pH of reaction mixture from ≤6 to ≥7.4 (Figure 2d). This is supported by a previous finding that showed no gelation in acidic pH whereas the gelation increased significantly with higher gel strength at basic pH.^[^ ^42^ ^]^

Cells are well known to respond to the mechanical properties of their environment.^[^ ^44^, ^45^, ^46^, ^47^ ^]^ Therefore, we evaluated the effect of prepolymer concentration on the mechanical properties of the hydrogels by varying the concentration of OFu‐Gel hydrogels from 5% to 15%. The compressive strength of the individual hydrogels was measured by determining their compressive moduli. The compressive strength increased significantly as the hydrogel concentrations increased: 5% OFu‐Gel hydrogels displayed an average compressive strength of 49 kPa, compared to 15% hydrogels, which displayed 150 kPa of compressive strength (Figure 2e). Similarly, we also analyzed the mechanical strength of the hydrogels by determining their storage moduli. We noted a significant increase in storage modulus of 0.965–4.681 kPa by increasing the prepolymer concentration from 5% to 15% (Figure S1, Supporting Information). These results highlight the tunability of the biomechanical properties of OFu‐Gel hydrogels.

To demonstrate that the fucoidan hydrogels could also be formed using a synthetic crosslinker instead of gelatin, we conducted a proof‐of‐concept study using 4‐arm polyethylene glycol (PEG) with terminal amine groups as a crosslinker. Indeed, mixing OFu with PEG‐amine resulted in hydrogel formation. We first evaluated the gelation parameters of OFu‐PEG hydrogels by varying the prepolymer concentrations and the ratios of OFu and 4‐arm PEG amine. We noted a similar gelation pattern as observed for OFu‐Gel hydrogels, i.e., the lowest concentration for hydrogel formation was 5% and an equal or higher proportion of 4‐arm PEG amine was required for complete gelation (Figure 2f). The OFu‐PEG hydrogels also demonstrated tunable mechanical strength as the compressive moduli increased significantly from 49 to 64 kPa in 5% and 15% OFu‐PEG hydrogels, respectively (Figure 2g). Similarly, the storage moduli also increased significantly from 0.811 to 5.956 kPa when increasing the prepolymer concentration of OFu‐PEG hydrogels from 5% to 15%, respectively (Figure S2, Supporting Information). Interestingly, the concentration of the hydrogel had a more pronounced effect on the mechanical strength of the OFu‐Gel hydrogel compared to OFu‐PEG hydrogels. This could be potentially due to the additional non‐covalent interactions between the fucoidan and gelatin, both of which are biopolymers with complex structures compared to PEG.

Together, these results demonstrated the development of a novel fucoidan hydrogel that can be crosslinked using amine‐containing molecules such as gelatin (natural polymer) or PEG (synthetic polymer). The imine‐crosslinked fucoidan hydrogels demonstrated tunable gelation time, pH responsiveness, and mechanical properties. Moreover, the degree of oxidation of fucoidan can be tuned by varying different parameters, such as molar ratio of sodium periodate, reaction time, and temperature,^[^ ^33^, ^34^ ^]^ which could further be leveraged to improve the tunability of the imine crosslinked fucoidan hydrogels.

### 2.2 Fucoidan Hydrogels Allow Light Transmission and Demonstrate Remarkable In Vitro Stability and Tunable Biodegradation Rate

**Figure 3:**
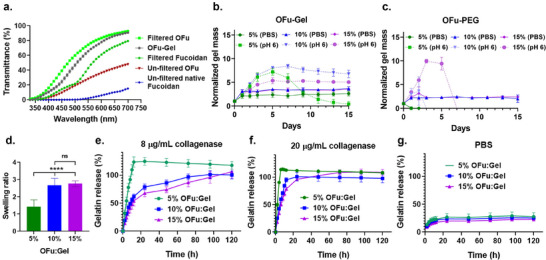
(a) Transmittance of native and oxidized fucoidan before and after filtration. (b) In vitro stability analysis of 5%, 10%, and 15% OFu‐Gel hydrogels in PBS and acidic medium (pH 6) monitored over a period of two weeks. (c) In vitro stability analysis of 5%, 10%, and 15% OFu‐PEG hydrogels in PBS and acidic medium (pH 6) monitored over a period of two weeks. The weight of hydrogels measured on each day was normalized by dividing it with the weight on day 0, taken prior to incubation in any medium and is referred to as “normalized gel mass”. (d) In vitro assessment of swelling ratio of 5%, 10%, and 15% hydrogels in PBS, p**** < 0.0001, one‐way ANOVA, Tukey's multiple comparisons test. (e–g) In vitro analysis of biodegradation pattern of 5%, 10%, and 15% OFu‐Gel hydrogels monitored over a period of 120 h when incubated in (e) 8 µg mL^−1^ collagenase enzyme, (f) 20 µg mL^−1^ collagenase enzyme, and (g) in PBS control. *n* = 3, mean ± SD for all.

One of the challenges in developing fucoidan‐based hydrogels is improving the light transmission required for imaging of cells cultured therein. To determine and compare the transparency of fucoidan before and after oxidation, a spectral scan was recorded from 250 to 700 nm using a Biotek Cytation 5 multimode plate reader. Native fucoidan exhibited very low transparency in the visible range, with negligible transmittance between 300 and 530 nm. Oxidation of native fucoidan slightly improved transparency compared to its unmodified form. However, filtration of native fucoidan using a vacuum filter unit with a 0.45 µm pore size significantly improved transparency, achieving ≈60–70% transmittance in the visible range. Subsequent oxidation of the filtered fucoidan further enhanced transmittance to ≈80–90%, indicating that both filtration and oxidation contribute to improve the overall transparency of fucoidan (**Figure** **3**a). The transparency of the OFu‐Gel hydrogels was similar to the filtered oxidized fucoidan, particularly at longer wavelengths (e.g., 600–700). Overcoming the limitation of light transmission is particularly important in fields such as in vivo imaging, real‐time monitoring of cell behavior, or studying cell spreading and migration in 3D environments, where clear visual observation is crucial for accurate analysis.^[^ ^48^, ^49^ ^]^ However, it is worth noting that the light transmission through fucoidan hydrogels is distinctive than the natural brown color of fucoidan which it acquires due to entrapment of pigments during isolation and extraction procedures.^[^ ^50^ ^]^ In conclusion, the increased transparency of filtered and oxidized fucoidan makes it highly suitable for applications requiring high optical transmission such as fluorescence imaging.

The stability and biodegradation profiles of hydrogels are crucial for optimizing their use in biomedical and tissue engineering applications, as these factors are critical for maintaining their structural integrity and supporting tissue growth. As OFu‐Gel and OFu‐PEG hydrogels are imine crosslinked hydrogels, they are prone to hydrolysis in an acidic environment.^[^ ^51^, ^52^ ^]^ Moreover, disease microenvironments are also known to be slightly acidic,^[^ ^53^, ^54^, ^55^ ^]^ thus, we assessed the in vitro stability of 5%, 10%, and 15% OFu‐Gel hydrogels in phosphate‐buffered saline (PBS) and in acidic environment with a pH 6, for two weeks. We observed a remarkable stability of all the tested concentrations of OFu‐Gel hydrogels in PBS for two weeks (Figure 3b). However, when incubated in an acidic environment, 5% OFu‐Gel hydrogels degraded completely within a period of two weeks while 10% and 15% remained stable, demonstrating their potential to be used in disease conditions (Figure 3b). We further extended the stability of OFu‐Gel hydrogels in PBS for a month and noticed a remarkable in vitro stability of these imine crosslinked hydrogels in PBS (Figure S3, Supporting Information). Considering the complex nature of interactions when gelatin is used as a crosslinker in OFu‐Gel hydrogels, we also evaluated the stability of imine crosslinked hydrogels using a synthetic crosslinker, 4‐arm PEG amine, in PBS and at acidic pH 6. We noted that while 5% OFu‐PEG hydrogels degraded within 2 days in PBS, both 10% and 15% OFu‐PEG hydrogels remained stable for two weeks. Interestingly, all the three tested concentrations of OFu‐PEG hydrogels degraded completely in an acidic environment due to dynamic nature of Schiff bonds present in the hydrogels (Figure 3c). The remarkable stability of imine crosslinked OFu‐Gel hydrogels compared to OFu‐PEG hydrogels could be due to a more complex chemical environment of the imine bonds, formed between OFu and gelatin.

An important consideration while developing biomaterials for in vivo applications is their swelling ratio to ensure that the hydrogel volume fits in the target cavity without exerting excessive pressure on the surrounding tissue.^[^ ^56^, ^57^ ^]^ To test this, we monitored the swelling of OFu‐Gel hydrogels after incubation in PBS and noticed lower swelling for 5% hydrogel (≈1.43X) compared to 10% (≈2.67X) and 15% hydrogels (≈2.76X) (Figure 3d). Since, these imine crosslinked hydrogels swell upon exposure to their environment, understanding the swelling behavior allows for a better control for in vivo administration.

A critical consideration of the injected or implanted biomaterials is their degradation rate, which is important for controlled drug release, new tissue formation, matrix remodeling, and efficient clearance from the body. In in vivo systems, matrix is remodeled through the highly controlled activity of numerous enzymes, including different classes of collagenases.^[^ ^58^, ^59^ ^]^ Here, we tested the biodegradation of OFu‐Gel hydrogel in the presence of collagenase‐I enzyme. We noted that polymer concentration was inversely proportional to the degradation rate: 5% OFu‐Gel hydrogels degraded ≈92% in 8 h in 8 µg mL^−1^ collagenase compared to ≈41% degradation observed in 15% hydrogels under the same conditions (Figure 3e). Moreover, the degradation rate was dependent on the collagenase concentration: 15% OFu‐Gel hydrogels degraded ≈96% in 20 µg mL^−1^ collagenase after 24 h of incubation, whereas the same hydrogel group degraded by ≈68% in 8 µg mL^−1^ collagenase‐I over the same period (Figure 3e,f). As expected, the hydrogels remained stable in control media (PBS without collagenase) (Figure 3g), indicating that the degradation was driven by the action of collagenase‐I.

Together, these findings demonstrated that the light transmission of OFu‐Gel hydrogels can be enhanced through filtration and oxidation. Additionally, these hydrogels exhibit stability in the in vitro environment and are also responsive to acidic environment and biodegradable in the presence of collagenase enzyme.

### 2.3 Imine Crosslinked Fucoidan Hydrogels are Self‐Healable

**Figure 4:**
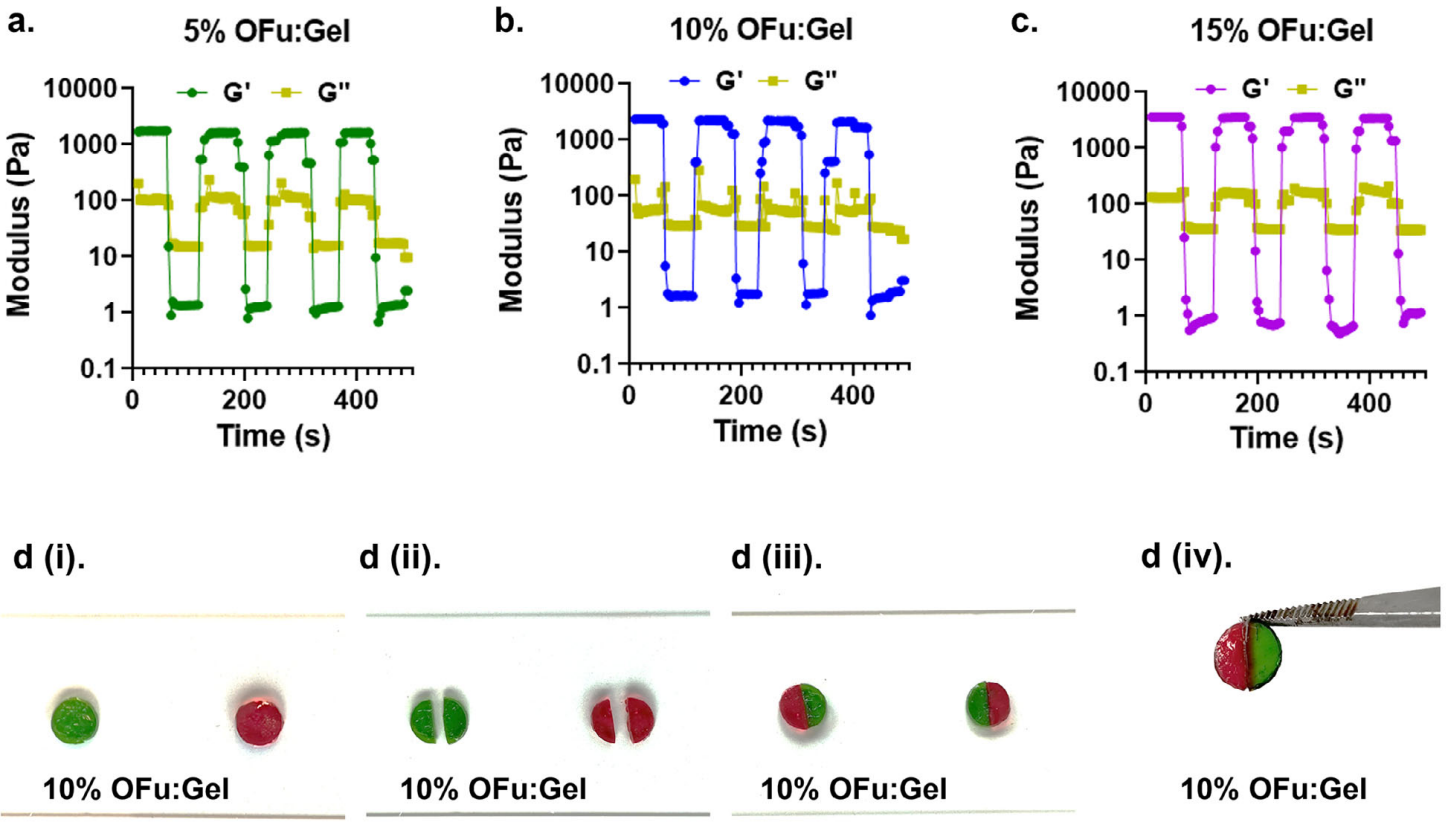
Rheological measurement of self‐healing of (a) 5%, (b) 10%, and (c) 15% OFu‐Gel hydrogels. (d) Qualitative visual observation of the self‐healing behavior of 10% OFu‐Gel hydrogels.

A major consideration when designing injectable hydrogels, is their self‐healing ability, that allows them to re‐crosslink after being subjected to mechanical forces during extrusion through a needle. A widely used approach to address this challenge is the introduction of reversible covalent crosslinks in the hydrogel network.^[^ ^60^, ^61^, ^62^ ^]^ We postulated that OFu‐Gel hydrogels would possess self‐healing property due to the presence of imine crosslinks in their hydrogel network, which are known to be reversible covalent crosslinks. To verify this, we subjected the fucoidan hydrogels (5%, 10% and 15%) to 8 steps of alternating low shear strain (0.1%) and high shear strain (1000%) using a rheometer. All tested concentrations of OFu‐Gel hydrogels demonstrated excellent self‐healing capabilities as evident with a significant shift in storage modulus (G′) and loss modulus (G″) between alternating steps of high and low shear strains (**Figure** **4**a–c). At 1000% shear strain, G″ was higher than the G′, indicating crosslinking disruption under high strain. Upon reducing the shear strain back to 0.1%, G′ exceeded G″, indicating the spontaneous re‐formation of reversible imine crosslinks resulting in instant gelation. This was further demonstrated by visual healing, where the 10% OFu‐Gel hydrogel disc was cut into two halves and reuniting these pieces led to their adhesion (Figure 4di–iv). The formation of a complete disc by reuniting these two halves is likely due to imine metathesis that led to the formation of new imine bonds between the two hydrogel pieces.^[^ ^63^ ^]^ These findings, for the first time, demonstrated the self‐healing capabilities of fucoidan hydrogels, thereby expanding the potential applications of fucoidan in tissue engineering.

### 2.4 Imine Crosslinked Fucoidan Hydrogels are Biocompatible and Support 2D Cell Culture

**Figure 5:**
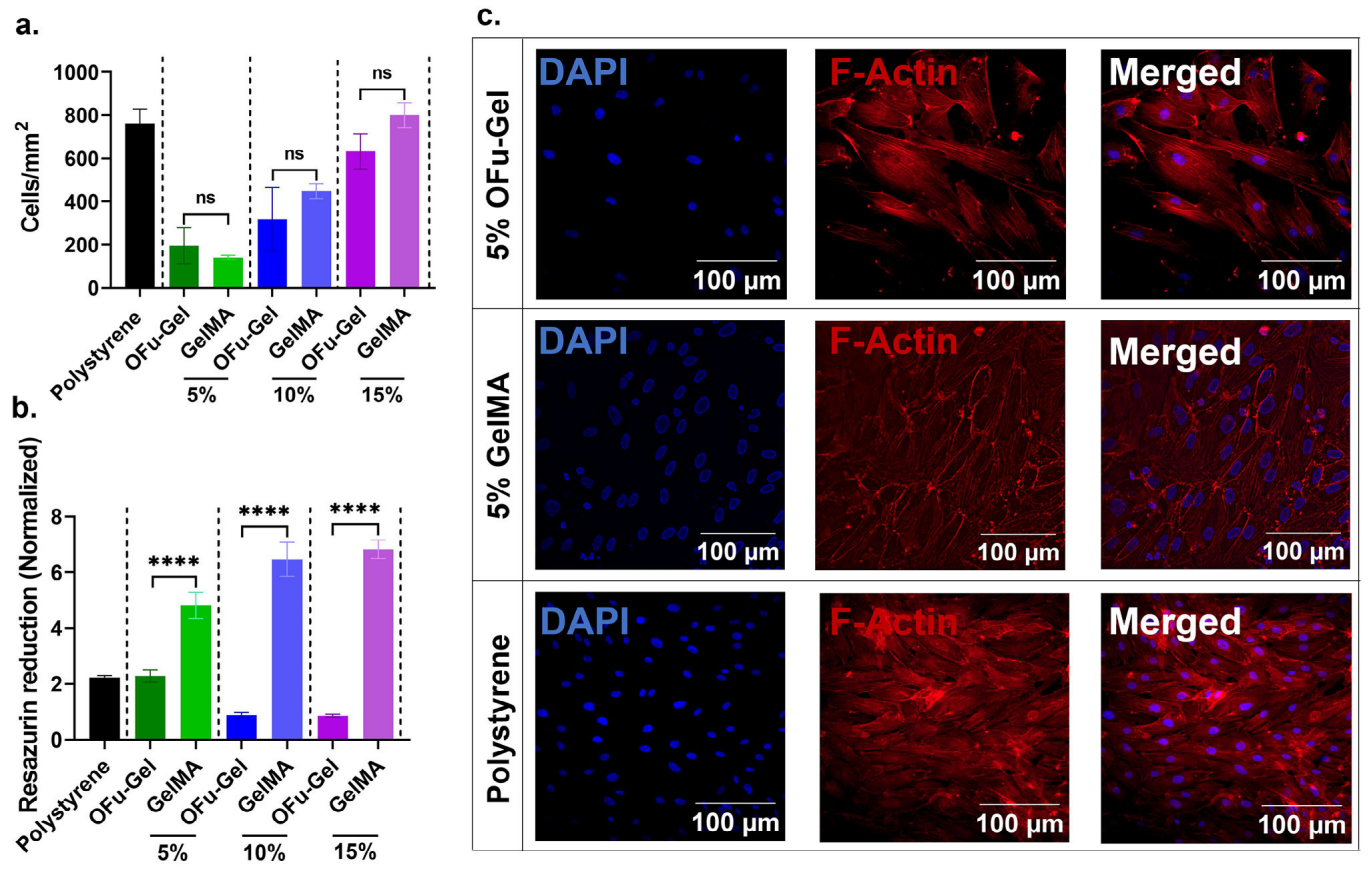
Biocompatibility of OFu‐Gel hydrogels in 2D cell culture. (a) TIME‐GFP cell adhesion after 3 h of initial seeding on 5%, 10%, and 15% OFu‐Gel and GelMA hydrogels, and polystyrene. (b) Fold change in metabolic activity of TIME‐GFP cells cultured on 5%, 10%, and 15% OFu‐Gel and GelMA hydrogels, and polystyrene observed on day 7 and normalized to metabolic activity monitored on day 1. p**** < 0.0001, one‐way ANOVA, Tukey's multiple comparisons test. (c) F‐Actin staining showing TIME‐GFP cell spreading on 5% OFu‐Gel, 5% GelMA and polystyrene. Scale bar represents 100 µm. *n* = 3, mean ± SD for all.

The biocompatibility of biomaterials is fundamental for their safe integration with biological systems. To test the in vitro biocompatibility of our hydrogels, we cultured hTERT immortalized GFP‐labelled microvascular endothelial (TIME‐GFP) cells on discs of 5%, 10%, and 15% OFu‐Gel hydrogels, with polystyrene and gelatin methacrylate (GelMA) used as controls. Cell adhesion to hydrogels of different stiffnesses was evaluated after 3 h of initial cell seeding. In general, the cell adhesion was higher as the prepolymer concentration of the hydrogels was increased from 5% to 15%, with more cells adhering to stiffer hydrogels. However, no statistically significant difference was observed in TIME‐GFP cell adhesion density on OFu‐Gel and GelMA hydrogels (**Figure** **5**a). Additionally, we monitored the viability of cultured TIME‐GFP cells over a week and observed similar metabolic activity between cells cultured on polystyrene and 5% OFu‐Gel hydrogels. In contrast, the metabolic activity of cells on 10% and 15% hydrogels remained similar to that observed on day 1, indicating lack of noticeable cell growth. Notably, metabolic activity of cells was significantly higher on the GelMA compared to OFu‐Gel hydrogel (Figure 5b). However, TIME‐GFP cells showed more spreading on 5% OFu‐Gel hydrogels compared to 5% GelMA and polystyrene controls (Figure 5c). Together, these data indicated that while cell adhesion and spreading on OFu‐Gel hydrogels is similar to GelMA, the cell growth is higher on GelMA hydrogels, potentially due to the higher gelatin content in GelMA hydrogels, which provides a more extracellular matrix‐like environment.

Surface coating of hydrogels with different proteins is known to improve cell functions such as cell adhesion, proliferation, and viability.^[^ ^64^ ^]^ Therefore, we coated the hydrogels with FNC coating mix (composed of fibronectin, collagen, and albumin) and assessed the cell adhesion and viability of TIME‐GFP cells. The cells exhibited a similar pattern of cell adhesion and viability as previously observed with cells cultured on uncoated hydrogels (Figure S4a,b, Supporting Information). Furthermore, no difference in cell spreading was observed between FNC‐coated OFu‐Gel hydrogel and the FNC‐coated polystyrene (Figure S4c, Supporting Information). In conclusion, OFu‐Gel hydrogels exhibited biocompatibility by supporting cell adhesion and spreading while maintaining cellular viability. This key characteristic highlights their non‐cytotoxic nature, making them suitable for tissue engineering applications.

### 2.5 Imine Crosslinked Fucoidan Hydrogels are Injectable and Support Neuronal Cell Differentiation

**Figure 6:**
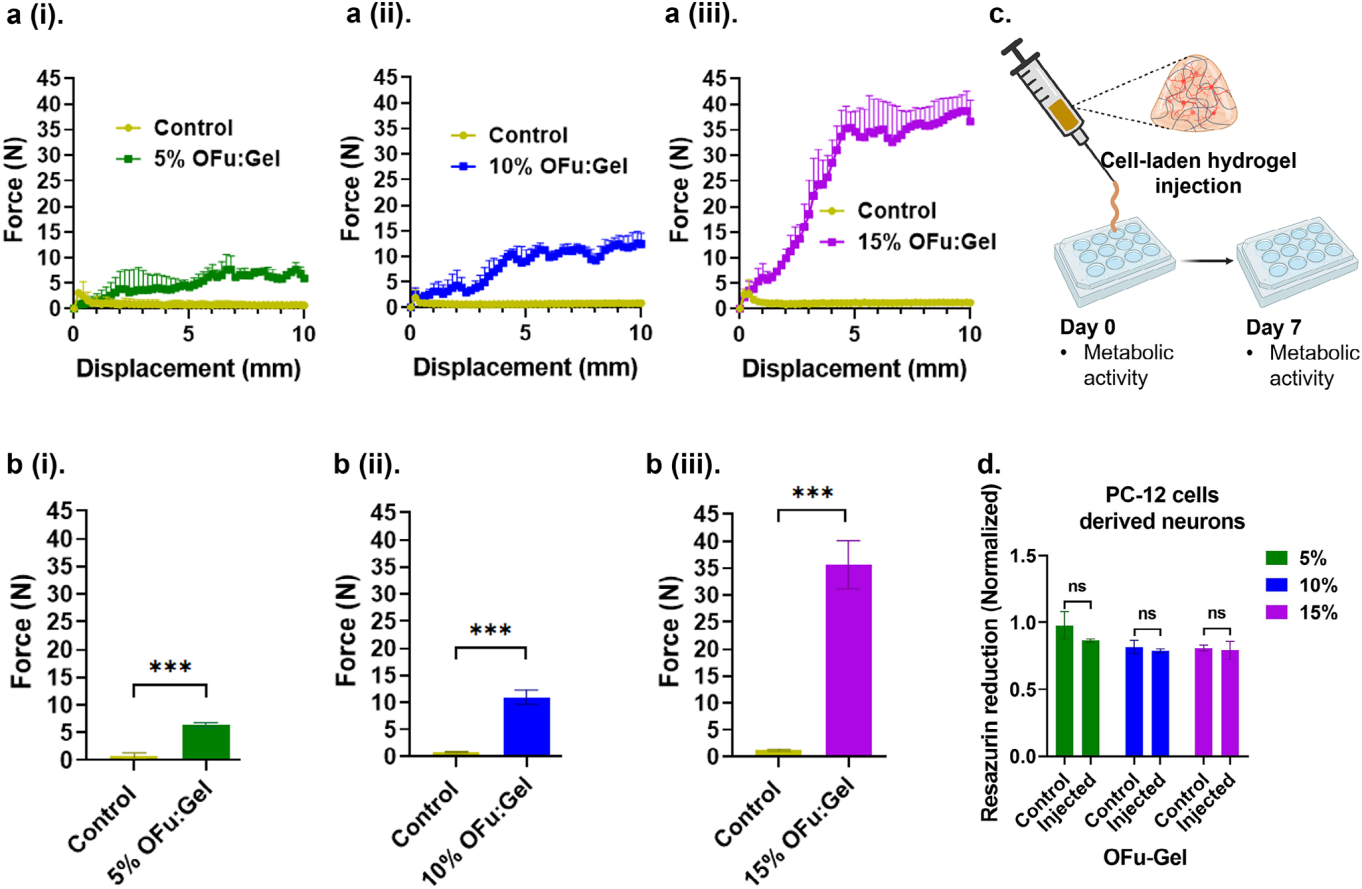
OFu‐Gel hydrogels are injectable and support cell viability. (a(i–iii)). Force (N) applied on (i) 5%, (ii) 10%, and (iii) 15% OFu‐Gel hydrogel as they were extruded through a 21G needle. (b(i–iii)). Force (N) required to extrude (i) 5%, (ii) 10%, and (iii) 15% OFu‐Gel hydrogel through a 21G needle, Student's *t*‐test, p*** < 0.001. (c) Schematic showing the experimental setup for cell‐laden hydrogel injection through 21G needle. (d) Fold change in metabolic activity of PC‐12‐derived neurons, encapsulated in 5%, 10%, and 15% OFu‐Gel hydrogels and extruded through 21G needle, monitored on day 7 and normalized to metabolic activity observed on day 1. Two‐way ANOVA, Sidak's multiple comparisons test. *n* = 3, mean ± SD for all.

Injectable hydrogels offer substantial advantages over implanted biomaterials for in vivo applications, including shape and size conformation of the target area while ensuring uniform interaction with the surrounding tissues.^[^ ^65^ ^]^ The external mechanical force applied during extrusion is not only detrimental to integrity of crosslinks but may also influence cellular viability. Therefore, we characterized the injection force required for extrusion of 5%, 10%, and 15% fucoidan hydrogels through a 21G needle. A customized syringe support was designed to firmly hold the syringe while the extrusion force was monitored using a Univert mechanical tester connected to a 100N load cell. Unmodified fucoidan mixed with gelatin (i.e., no imine crosslinking) was used as a control. As expected, a significant increase in the extrusion force was noted for all the three tested hydrogel groups compared to their respective control groups (**Figure** **6**a(i–iii)). Moreover, as the polymer concentration in the hydrogels increased from 5% to 15%, a significant increase in the extrusion force from 6.4 to 35.6 N was observed, due to the higher crosslinking density and greater mechanical strength of the 15% OFu‐Gel hydrogels compared to the 5% hydrogel (Figure 6b(i–iii)). The higher crosslinking density led to a significant increase in the external mechanical force of extrusion required to extrude the hydrogel through the 21G needle.

To test if OFu‐Gel hydrogels support the injection of cell‐laden hydrogels, we first evaluated their compatibility with neuronal progenitor (PC‐12) cells. Soluble fucoidan has shown potential to improve neural regeneration.^[^ ^66^ ^]^ Therefore, we evaluated whether OFu‐Gel hydrogels would support the differentiation of PC‐12 cells into mature neurons. To test this, we cultured PC‐12 cells on discs of 5%, 10%, and 15% OFu‐Gel hydrogels, with and without collagen coating, in Nerve Growth Factor (NGF) supplemented differentiation media for a week. After 7 days of culture on OFu‐Gel hydrogels, cells were stained with β‐III tubulin, which revealed that 15% fucoidan hydrogels facilitated the highest level of neuronal differentiation compared to the 5% and 10% OFu‐Gel groups (Figure S5, Supporting Information). Moreover, after 7 days of differentiation, no difference in cell viability was observed between collagen‐coated and uncoated hydrogels (Figure S6, Supporting Information). This consistent cell viability, similar to that observed on day 1, could be due to the differentiation of the cells into neurons, which inhibits cell proliferation.^[^ ^67^ ^]^

Subsequently, we encapsulated the PC‐12‐derived neurons in OFu‐Gel hydrogels (5%, 10%, and 15%), injected the cell‐laden hydrogels through 21‐gauge needle, and monitored post‐injection viability of encapsulated cells for over 1 week (Figure 6c). We observed a slight decrease in metabolic activity; however, no statistically significant difference in cell viability was observed between the injected and control groups (Figure 6d). Furthermore, as NGF treatment halts the replication of PC‐12 neuronal cells, it is worth noting that this results in stable metabolic activity over time.^[^ ^67^ ^]^ Together these findings suggest that the extrusion force depends on the polymer content in the fucoidan hydrogel. However, the force required to extrude 5%, 10%, and 15% OFu‐Gel hydrogels did not impact the viability of encapsulated PC‐12‐derived neurons.

### 2.6 Imine Crosslinked Fucoidan Hydrogels Possess In Vitro and In Vivo Immune‐Modulatory Properties

**Figure 7:**
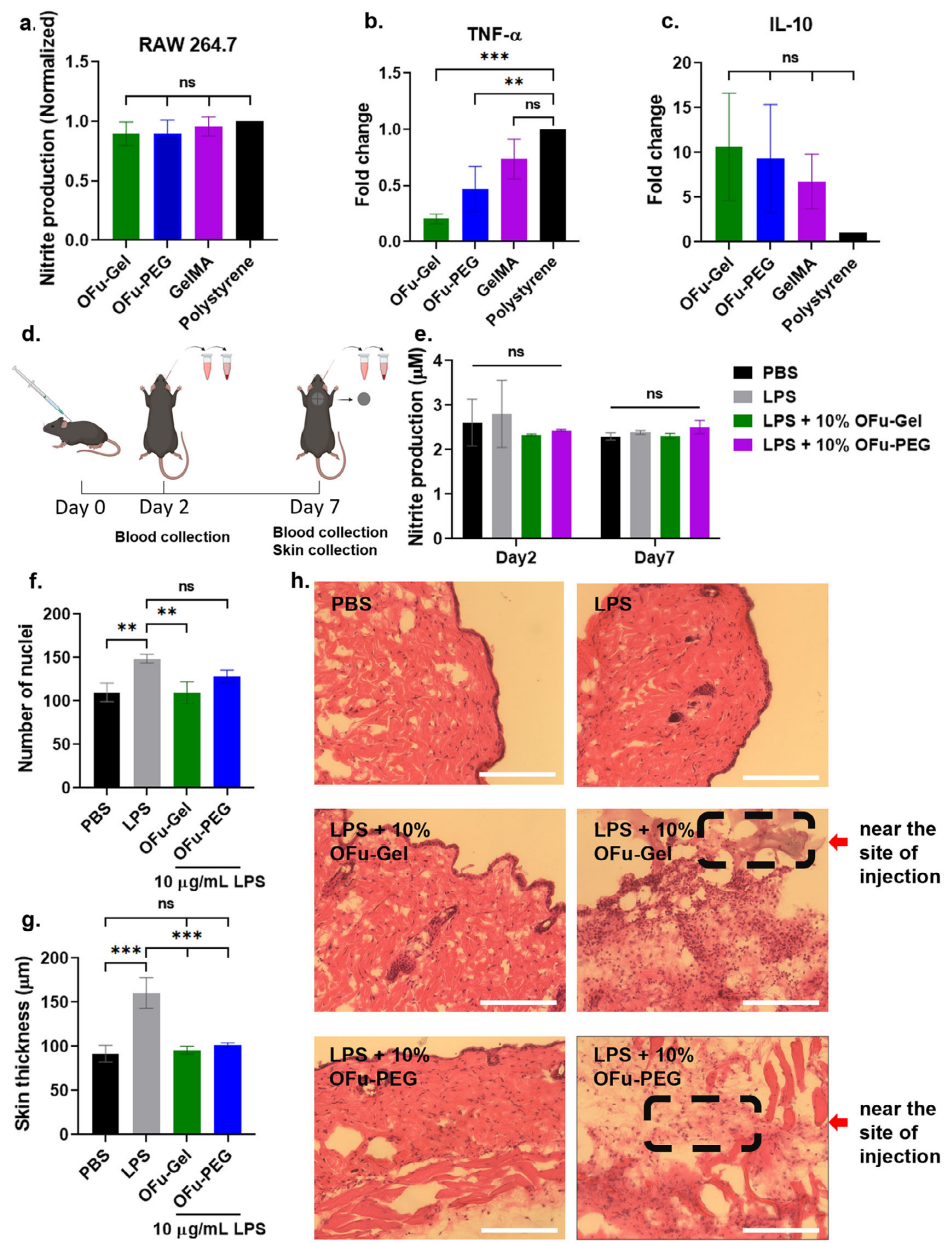
Fucoidan hydrogels demonstrated immunomodulatory properties. (a) Quantification of nitrite production by LPS activated RAW 264.7 cells cultured on 10% OFu‐Gel, 10% OFu:PEG, 10% GelMA and polystyrene, one‐way ANOVA, Tukey's multiple comparisons test. (b,c) Fold change in (b) TNF‐α and (c) IL‐10 secretion by RAW 264.7 cells cultured on 10% OFu‐Gel, 10% OFu:PEG, 10% GelMA and polystyrene after LPS activation. p*** < 0.001, p** < 0.01, one‐way ANOVA, Tukey's multiple comparisons test. (d) Schematic of experimental plan to assess the biocompatibility of OFu hydrogels in C57BL/6 mice model. 10% OFu‐Gel and 10% OFu:PEG hydrogels were injected subcutaneously in mice. Blood samples were withdrawn on days 2 and 7. Mice were euthanized for collecting skin samples on day 7 for histological analysis. (e) Quantification of nitrite levels from mice serum detected on days 2 and 7, two‐way ANOVA, Sidak's multiple comparisons test, *n* = 3, mean ± SD for all. (f) Number of nuclei counted from epidermal layer of skin sections. (g) Epidermal thickness of skin sections in µm, p*** < 0.001, one‐way ANOVA, Tukey's multiple comparisons test. (h) H&E staining of mice skin sections harvested after 7 days of subcutaneous injection of hydrogels. Rectangular section showing the site of injection of hydrogel, scale bar represents 200 µm, *n* = 3.

Fucoidan has recently gained prominence due to its significant immunomodulatory and anti‐inflammatory effects, which have been leveraged in tumor immunotherapy.^[^ ^68^, ^69^ ^]^ Fucoidan influences the levels of inducible nitric oxide synthase (iNOS), thereby reducing the inflammatory responses.^[^ ^70^, ^71^ ^]^ In order to evaluate the anti‐inflammatory properties of fucoidan hydrogel, we cultured RAW 264.7 macrophages on 10% fucoidan hydrogels crosslinked using both synthetic 4‐arm PEG amine crosslinker (OFu‐PEG) and natural gelatin crosslinker (OFu‐Gel) and compared the macrophage markers to those cultured on widely used gelatin methacrylate hydrogel (10% GelMA) with polystyrene as a control. After 24 h, RAW 264.7 cells were activated with 10 µg mL^−1^ lipopolysaccharide (LPS), and 48 h later, the nitrite levels were monitored using the Griess assay. The nitrite levels slightly decreased in fucoidan hydrogels compared to GelMA and polystyrene control in LPS‐treated cells (**Figure** **7**a). Next, we quantified the expression of well‐known pro‐inflammatory cytokine Tumor Necrosis Factor‐α (TNF‐α), and anti‐inflammatory cytokine Interleukin‐10 (IL‐10) using ELISA. Both the OFu‐Gel and OFu‐PEG fucoidan hydrogels significantly decreased TNF‐α levels compared to polystyrene group (*p* = 0.0004 and 0.0059, respectively) (Figure 7b). Furthermore, IL‐10 levels were increased in OFu‐Gel (*p* = 0.1153) and OFu‐PEG (*p* = 0.1890) hydrogel groups compared to polystyrene group (Figure 7c). Soluble fucoidan is known to downregulate TNF‐α and upregulate IL‐10^[^ ^72^ ^]^ in LPS‐stimulated macrophages. Notably, our findings indicated that fucoidan retained its anti‐inflammatory properties even after being chemically modified and crosslinked to form fucoidan hydrogels, thereby facilitating its potential use as an immunomodulatory scaffold for tissue engineering applications.

To test whether the fucoidan hydrogels are biocompatible in vivo, 10% OFu‐Gel and 10% OFu‐PEG hydrogels were subcutaneously injected into C57BL/6 mice. An intraperitoneal dose of 10 µg mL^−1^ LPS was administered prior to subcutaneous injection of fucoidan hydrogels. PBS‐only and LPS‐only groups were used as controls. The inflammatory response was monitored from blood plasma by detecting the nitrite levels on days 2 and 7 (Figure 7d). The results suggested no difference in nitrite levels between the experimental and control groups on both day 2 and day 7. However, on day 2, slight inflammation was observed in the LPS group compared to OFu hydrogel groups (Figure 7e). The similar levels of nitrite among the groups receiving LPS + OFu based hydrogels and PBS control group indicated the ability of fucoidan to downregulate the iNOS levels and subsequently decrease nitric oxide production, thus reducing the inflammation.^[^ ^70^ ^]^ Additionally, histological examination was performed on skin samples (from the site of hydrogel injection) collected on day 7 to observe any difference in cellular infiltration due to hydrogel injection and LPS‐induced systemic inflammation. It is well‐known that LPS administration leads to a systemic inflammation, causing inflammatory cells to infiltrate the skin.^[^ ^73^ ^]^ To examine the inflammatory cells infiltration, we compared the number of nuclei present in the epidermal skin layer of the different mice groups. Notably, the LPS + OFu‐Gel (*p* = 0.0039) and PBS (*p* = 0.0041) groups displayed significantly lower cellular infiltration compared to the LPS‐only group. Additionally, LPS + OFu‐PEG also displayed a decrease in cellular infiltration; however, it was not statistically significant compared to LPS‐only group (*p* = 0.1006) (Figure 7f). Inflammation is known to cause an increase in skin thickness due to infiltration of immune cells and edema. We quantified the skin thickness of all four mice groups and observed a significantly lower skin thickness in the LPS + OFu‐Gel (p = 0.0003), LPS + OFu‐PEG (*p* = 0.0005) and PBS (*p* = 0.0002) groups compared to the LPS only group, further complimenting the anti‐inflammatory role of fucoidan hydrogels (Figure 7g). Moreover, H&E staining revealed the conservation of skin architecture, and no signs of inflammation and bleeding were found at the site of injection of OFu hydrogels (Figure 7h).

To dissect the in vivo inflammatory response against the injected OFu hydrogels, skin tissue samples collected from the site of injection were stained with antibodies specific for pro‐inflammatory (iNOS and TNF‐α) and anti‐inflammatory (CD‐206 and arginase‐I) markers.^[^ ^74^, ^75^, ^76^, ^77^, ^78^, ^79^ ^]^ The immunohistochemistry data revealed attenuation of expression of pro‐inflammatory (iNOS and TNF‐α) markers in OFu hydrogel groups compared to LPS control (Figure S7, Supporting Information). Moreover, the expression of anti‐inflammatory (CD‐206 and arginase‐I) markers increased in OFu hydrogel groups compared to LPS control group (Figure S8, Supporting Information). This confirms the in vivo immunomodulatory property of fucoidan by suppressing the systemic inflammatory responses and creating an anti‐inflammatory environment at the site of injection. Together these findings indicated the in vivo biocompatibility and anti‐inflammatory nature of fucoidan hydrogel. In conclusion, both in vitro and in vivo experiments demonstrate that fucoidan hydrogels naturally reduce inflammatory responses without the need for additional cytokines to modulate the microenvironment. Future studies will focus on utilizing fucoidan hydrogels for the therapeutic delivery of neuronal progenitor cells to enhance in situ neuronal tissue regeneration.

## 3 Conclusion

This study successfully developed injectable fucoidan‐based hydrogels with tunable physicochemical properties and significant immunomodulatory potential without the need of introducing any immune‐regulatory adjuvants. By introducing aldehyde groups into fucoidan through periodate oxidation and crosslinking with amine‐containing polymers, reversible imine‐crosslinked hydrogels were formed. These hydrogels demonstrated tunable biomechanical features, self‐healing ability, and long‐term stability, countering the typical instability of imine bonds. Additionally, their injectability and biocompatibility make them suitable for cell encapsulation and culture. In vitro and in vivo experiments confirmed the immune‐modulatory ability of fucoidan hydrogel, demonstrating their ability to reduce inflammation and creating a pro‐regenerative environment. This work positions fucoidan hydrogels as a promising platform for therapeutic applications in cell transplantation and tissue engineering, addressing critical challenges in regenerative medicine.

## 4 Experimental Section

### Oxidation of Fucoidan

Fucoidan derived from *Fucus vesiculosus* (Marinova Inc.) was oxidized using sodium periodate as described previously.^[^ ^31^, ^80^ ^]^ Briefly, 3 g of powdered fucoidan were dissolved in 300 mL of deionized water. Sodium periodate (NaIO₄) was then added in 2.5 molar excess to the aqueous fucoidan solution, and the reaction was allowed to run in dark for 8 h at room temperature with continuous stirring. Following the reaction, 60 mL of ethylene glycol was added to terminate the oxidation process. The resulting reaction mixture was subjected to dialysis for 5 days to remove any unreacted compounds, using a dialysis membrane with a molecular weight cutoff of 12–14 kDa. Dialysis water was refreshed three times on the first day, and twice daily from the second to the fifth day. The oxidized fucoidan solution was then purified via vacuum filtration unit with a 0.45 µm pore size. The filtrate was subsequently freeze‐dried and stored at −20 °C until further use.

The degree of oxidation of fucoidan was determined through hydroxylamine hydrochloride titration method.^[^ ^81^ ^]^ Briefly, 25 mg of OFu was dissolved in 6.25 mL of 0.25 N hydroxylamine hydrochloride methyl orange solution and incubated in dark for 2 h. Titration was carried out after incubation using 1 M sodium hydroxide solution. The amount of sodium hydroxide required to attain red to yellow endpoint was noted and the following formula was used to determine the degree of oxidation of native fucoidan.^[^ ^81^ ^]^

(1) $$\begin{array}{ccc} & & \mathrm{Degree}\;\mathrm{of}\;\mathrm{oxidation}\\ & & \;=\frac{\mathrm{MW}\;\mathrm{of}\;\mathrm{fucoidan}\;x\;\mathrm{volume}\;\mathrm{of}\;\mathrm{NaOH}\;x\;\mathrm{concentration}\;\mathrm{of}\;\mathrm{NaOH}}{2\;x\;\mathrm{weight}\;\mathrm{of}\;\mathrm{sample}} \end{array}$$

### Synthesis of Gelatin‐Methacrylate (GelMA)

GelMA was synthesized as reported previously.^[^ ^82^ ^]^ Briefly, 10 g of gelatin (Type A) was dissolved in 100 mL of carbonate‐bicarbonate buffer at 50 °C. Methacrylic anhydride (1 mL) was added, and the reaction was run for 2 h with constant stirring. The reaction was terminated by adjusting the pH to 7.4. The mixture was diluted, filtered, and dialyzed for 3 days at 37 °C. After dialysis, the solution was frozen and lyophilized. The degree of substitution was 90%, quantified by 2,4,6‐Trinitrobenzene Sulfonic Acid (TNBS) assay. GelMA hydrogels were made by dissolving lyophilized GelMA in PBS containing 0.5% w/v Irgacure 2959.

### Rheological Characterization

The rheological characterization of hydrogels was performed by using rheometer (ThermoScientific^TM^ HAAKE^TM^ MARS^TM^ 60 Rheometer). Briefly, 5%, 10%, and 15% hydrogel samples were prepared by mixing OFu and gelatin in 1:1 ratio. The polymer solutions were pipetted in a custom‐made glass chamber and the samples were incubated in a humidified environment at 37 °C for an hour. Oscillation frequency sweep was performed on OFu hydrogels by applying a constant shear strain of 1% at 37 °C and measuring the storage and loss moduli over a frequency range of 0.1–100 Hz. The self‐healing of the hydrogels was evaluated by subjecting the samples to alternating shear strains from 0.1% to 1000% over eight intervals of 1 min each. All measurements were performed using a 20 mm parallel plate geometry, with data collected from three individual samples for each hydrogel formulation to ensure reproducibility.

### Mechanical Characterization

For mechanical characterization, OFu and gelatin solutions prepared in phosphate‐buffered saline (PBS) were mixed in 1:1 ratio, with a total polymer concentration of 5%, 10%, and 15%. The prepolymer solutions were pipetted into a glass chamber with a thickness of 1 mm. The samples were allowed to gel in a humidified environment at 37 °C, and hydrogel discs of 6 mm diameter were collected by punching the hydrogels using a biopsy punch. To determine their mechanical strength, the hydrogels were subjected to mechanical compression using the CellScale Univert mechanical tester connected to a 10N load cell, as previously described.^[^ ^83^ ^]^ Briefly, the initial contact force of 0.1 N was applied to determine the hydrogel surface. Subsequently, a 10% compression was applied at a rate of 0.5 mm min^−1^ to flatten the hydrogel surface. Mechanical strength was assessed by applying an additional 10% compression, in five steps of 2% compression each. The resulting data was used to generate stress‐strain curves for each of the five compression steps. The compressive strength of each hydrogel sample was calculated by analyzing and averaging the slope of these stress‐strain curves. The compressive strength of three individual samples per group were determined for statistical analysis.

### Biodegradation

For biodegradation study, OFu‐Gel hydrogels were directly prepared in the wells of 96 well plates by pipetting 60 µL of OFu‐Gel polymer solution. Briefly, the hydrogels were incubated at 37 ⁰C in 200 µL of PBS (control), and collagenase‐I solution at a concentration of 8 and 20 µg mL^−1^ prepared in PBS. To observe the rate of biodegradation, the solutions from each well were periodically collected and replaced with fresh enzyme solution. The released gelatin protein, which acted as a crosslinker, was used as a proxy for hydrogel degradation and was quantified using BCA protein assay kit according to the manufacturer's instructions. The protein concentrations were quantified by referencing it with a calibration curve and the rate of degradation of the hydrogels was monitored over time.

### Swelling and Stability Testing

To test the stability of the OFu hydrogels in neutral and acidic environments, 5%, 10%, and 15% hydrogel samples were prepared and incubated at 37 ⁰C in PBS and in acidic environment with pH 6 for a period of 2 weeks. The weight of the hydrogels was measured before incubation in the PBS and acidic medium and was regarded as weight (s) on “Day 0″. The hydrogels were incubated in PBS and acidic medium (pH 6) and their stability was monitored for 2 weeks by measuring the wet weight (w) every other day. The weight of the hydrogels measured on different days was normalized with the weight of hydrogels on “Day 0″ (prior to incubation in any medium) and the data was represented as “normalized gel mass” over time. For determining the swelling of the hydrogel samples, the maximum wet weight (w) attained by the hydrogels during the incubation period was noted, and the following formula was used to calculate the swelling ratio of the hydrogels.^[^ ^84^ ^]^

(2) $$\mathrm{Swelling}\;\mathrm{ratio}=\frac{\mathrm{Weight}\;\left(w\right)-\mathrm{Weight}\;\left(s\right)}{\mathrm{Weight}\;\left(s\right)}$$

### Injectability Test

The force required to extrude 5%, 10%, and 15% OFu‐Gel hydrogels through a 21G needle was measured using a CellScale Univert mechanical tester connected with a 100 N load cell, adapted to hold a 1 mL syringe containing the hydrogel samples. Extrusion was performed at a constant rate of 1 mm s^−1^, and the force exerted on the syringe plunger was recorded. For comparison, control groups were established using unmodified native fucoidan mixed with gelatin solution, and the extrusion force for the respective control groups was also measured. The average extrusion force for each hydrogel concentration was calculated based on data collected from three independent samples per group.

### 2D Culture of TIME‐GFP and PC‐12 Cells

Microvascular endothelial cells (TIME‐GFP cells, ATCC) and neuronal progenitor cells (PC‐12, ATCC), were cultured in their respective recommended media within a humidified incubator at 37 °C. Upon reaching ≈85% confluency, the cells were trypsinized and seeded onto OFu‐Gel and GelMA hydrogel discs at densities of 10000 cells cm^−^^2^ for TIME‐GFP cells and 40000 cells cm^−^^2^ for PC‐12 cells. To assess the influence of surface coatings on cell adhesion, viability, and spreading, OFu hydrogels were pre‐coated with FNC coating mix (Thermofisher Scientific) for 20 min prior to seeding TIME‐GFP cells. Cell adhesion of TIME‐GFP cells was evaluated by replacing the media with fresh medium after 3 h and counting the cells that adhered to the hydrogels. Additionally, the metabolic activity of TIME‐GFP cells was assessed on days 1 and 7 using resazurin assay (Biotium), following the manufacturer's instructions.

### Differentiation of PC‐12 Cells

To promote the adhesion of PC‐12 cells to the hydrogels, the hydrogel samples were pre‐coated with collagen type I derived from rat tail at a concentration of 6 µg cm^−^^2^. For inducing neuronal differentiation, PC‐12 cells were cultured in differentiation media supplemented with 50 ng mL^−1^ nerve growth factor (NGF, Alomone Labs).^[^ ^85^ ^]^ The cells were allowed to differentiate and develop neuronal projections over the course of 1 week, with media changes occurring every other day. The metabolic activity of the PC‐12 cells was assessed on days 1 and 7. Following the differentiation, the cells were fixed and stained with anti‐β III tubulin antibody to visualize the axonal projections of the differentiated neurons as described under “*Immunochemistry*” section.

### Effect of Injectability on Metabolic Activity of PC‐12 Cells

To assess the impact of injectability on PC‐12 cell viability, differentiated PC‐12 cells were encapsulated within OFu hydrogels with the concentrations of 5%, 10%, and 15%. These hydrogels were then extruded through a 21G needle to simulate the injection process. For comparison, PC‐12 cells were cultured in 3D hydrogels that did not undergo extrusion, serving as controls. The metabolic activity of PC‐12 cells within the hydrogels was assessed on days 1 and 7 to determine the effect of the mechanical forces associated with extrusion on cell viability.

### Immunochemistry

After a 7‐day culture of TIME‐GFP and PC‐12 cells, the cells were fixed overnight using 4% paraformaldehyde (PFA) for TIME‐GFP cells and 2% PFA for PC‐12 cells. Following fixation, the cells were permeabilized with 0.5% Triton X‐100 and blocked with 3% bovine serum albumin (BSA) for 1 h. To visualize the F‐Actin network in TIME‐GFP cells, the cells were incubated with Phalloidin Alexa Fluor 647 for 1 h. For β‐III tubulin staining in differentiated PC‐12 cells, the cells were incubated overnight at 4 °C with anti‐β III Tubulin primary antibody (Abcam, ab7751) followed by incubation with an anti‐mouse IgG Alexa Fluor 546 secondary antibody (Invitrogen, A11018), overnight at 4 °C. Nuclei were counterstained with Hoechst (Invitrogen, H3570) for 1 h prior to imaging. After each immunostaining step, the cells were washed thrice with 0.05% Tween 20 in PBS for 30 min per wash. The stained samples were imaged using an AxioObserver fluorescent microscope, and the images were analyzed using ImageJ software.

To stain the cryosections with iNOS, TNF‐α, CD‐206, and arginase‐ I specific antibodies, the cryosections of skin tissue were defrosted and fixed with chilled acetone for 10 mins. Subsequently, the sections were permeabilized and blocked in animal free blocking buffer using 0.5% Triton‐X and 3% BSA, respectively for 45 min. Prior to using mouse primary antibodies, sections were blocked by using goat F(ab) Anti‐Mouse IgG H&L (Abcam) overnight at 4 °C. The sections were subsequently stained with primary antibodies specific for iNOS, TNF‐α, CD‐206, and Arginase‐I (detailed information about antibodies is given in Table S2, Supporting Information) by incubating them overnight at 4 °C, followed by incubating them with secondary antibodies overnight at 4 °C. Sections were washed thrice after each step of staining protocol and were mounted with Vectashield + DAPI (nuclear stain) and were covered with a coverslip. The stained sections were imaged using an AxioObserver fluorescent microscope, and the images were analyzed using ImageJ software.

### Enzyme‐Linked Immunosorbent Assay (ELISA)

ELISA was performed to quantify the pro‐inflammatory cytokine (TNF‐α) and anti‐inflammatory cytokine (IL‐10) secreted by RAW 264.7 cells cultured on OFu‐based hydrogels. Briefly, RAW 264.7 cells were cultured on 10% OFu‐Gel and 10% OFu‐PEG hydrogels, polystyrene and 10% GelMA, a widely used hydrogel biomaterial, as a control, at a density of 10000 cells per cm^2^ for 24 h. After 24 h, cells were treated with 10 µg mL^−1^ LPS and cultured for 48 h. The cell supernatant was collected from LPS‐treated and untreated samples after 2 days of incubation and the cytokine release was determined using ELISA cytokine detection kits for TNF‐α and IL‐10 according to the manufacturer's instructions (Chondrex Inc.). The cytokine levels were quantified by referencing it with a calibration curve.

### Nitrite Production Assay

Griess assay was performed to quantify the nitrite production both in vitro and in vivo. Briefly, RAW 264.7 cells were cultured on OFu‐Gel, OFu‐PEG, GelMA hydrogels and polystyrene, as described above. The cell supernatant was collected for quantifying the nitrite levels. Nitrite was also quantified from in vivo plasma samples collected from mice on days 2 and 7 by referencing it with a calibration curve according to the manufacturer's protocol (Promega).

### Subcutaneous Injections of Hydrogels in C57BL/6 Mice

A proof‐of‐concept in vivo study was performed to evaluate the performance of fucoidan hydrogels in mice. The experimental procedures involving animals were approved by the Institutional Animal Care and Use Committee (IACUC) of the Michigan Technological University (# L‐0317). Twelve wild‐type 8‐week‐old C57BL/6 male mice (Purchased from The Jackson Laboratory) were used in the experiments. The mice were kept at the animal facility with constant humidity and room temperature under controlled light and dark cycle. To evaluate the in vivo biocompatibility and immunomodulatory properties of the fucoidan hydrogel, the mice were randomly divided into 4 groups: PBS only; 10 µg mL^−1^ LPS only; 10 µg mL^−1^ LPS + 10% OFu‐Gel injection; and10 µg mL^−1^ LPS + 10% OFu‐PEG injection. All the mice were anesthetized with isoflurane before giving intraperitoneal dose of 10 µg mL^−1^ LPS and subcutaneous injection of respective hydrogels on day 0. Blood samples were collected on days 2 and 7 to assess the inflammatory response by quantifying nitrite levels from blood plasma. All the mice were euthanized on day 7 and skin samples from the site of injection were collected for further evaluation.

### Hematoxylin and Eosin (H&E) Staining

All the skin samples were immediately fixed overnight in 4% paraformaldehyde and were subsequently dehydrated in 10%, 20%, and 30% sucrose solutions before embedding in OCT compound. 10 µm thick cryosections of the embedded skin tissue samples were prepared using a cryostat. For H&E staining of the cryosections, the OCT compound was first removed by immersing and washing the tissue sections in xylene twice for 2 min. Subsequently, the sections were dehydrated through two rounds of 2‐min washes in absolute ethanol and 95% ethanol. After dehydration, the sections were rinsed with distilled water and stained with hematoxylin solution for 50 s. Excess hematoxylin was washed off with distilled water, and the tissue was differentiated using 1% acid alcohol for 1 min. Following differentiation, sections were counterstained with eosin Y for 20 s, then dehydrated again through two rounds of 10‐s washes in 95% ethanol and absolute ethanol. Finally, the stained sections were cleared in xylene, mounted for imaging using light microscope.

### Statistical Analysis

Statistical analysis was performed using GraphPad PRISM 8.0.2. All the data is presented as mean ± SD with at least three replicates. For comparison of two groups, a Student's *t*‐test was used. For more than two dependent variable groups, One‐way ANOVA was performed with Tukey's multiple comparisons test. For comparison of more than two dependent variable groups with two independent variables, Two‐way ANOVA was performed. A *p*‐value which is ≤ 0.05 was considered as statistically significant.

## Conflict of Interest

The authors declare no conflict of interest.

## Supporting information



Supporting Information

## Data Availability

The data that support the findings of this study are available in the supplementary material of this article.

## References

[1] A. Citkowska , M. Szekalska , K. Winnicka , Marine Drugs 2019, 17, 458. 31387230 10.3390/md17080458 PMC6722496

[2] K. N. Ekdahl , J. D. Lambris , H. Elwing , D. Ricklin , P. H. Nilsson , Y. Teramura , I. A. Nicholls , B. Nilsson , Adv. Drug Delivery Rev. 2011, 63, 1042. 10.1016/j.addr.2011.06.012 PMC3166435 21771620

[3] M. B. Gorbet , M. V. Sefton , Biomater.: Silver Jubilee Compendium 2004, 25, 219.

[4] C. J. Wilson , R. E. Clegg , D. I. Leavesley , M. J. Pearcy , Tissue Eng. 2005, 11, 1. 15738657 10.1089/ten.2005.11.1

[5] H.‐C. Yang , H. C. Park , H. Quan , Y. Kim , Biomimetic Medical Materials: From Nanotechnology to 3D Bioprinting, Springer, Singapore 2018 pp. 197–206.

[6] C. Esche , C. Stellato , L. A. Beck , J. Invest. Dermatol. 2005, 125, 615. 16185259 10.1111/j.0022-202X.2005.23841.x

[7] J. M. Anderson , A. Rodriguez , D. T. Chang , Semin. Immunol 2008, 20, 86. 18162407 10.1016/j.smim.2007.11.004 PMC2327202

[8] L. Perry , F. Karp , K. Hauch , B. D. Ratner , J. Undergrad. Res. Bioeng. 2007, 4, 11.

[9] B. G. Keselowsky , A. W. Bridges , K. L. Burns , C. C. Tate , J. E. Babensee , M. C. LaPlaca , A. J. García , Biomaterials 2007, 28, 3626. 17521718 10.1016/j.biomaterials.2007.04.035 PMC1950471

[10] J. Ohmes , L. M. Saure , F. Schütt , M. Trenkel , A. Seekamp , R. Scherließ , R. Adelung , S. Fuchs , Marine Drugs 2022, 20, 402. 35736205 10.3390/md20060402 PMC9229026

[11] F.‐e. Ettoumi , H. Huang , Y. Xu , L. Wang , Q. Ru , Y. Hu , L. Zou , Z. Luo , Food Hydrocoll. 2024, 154, 110108.

[12] D. He , A. S. Zhao , H. Su , Y. Zhang , Y. N. Wang , D. Luo , Y. Gao , J. A. Li , P. Yang , J. Biomed. Mater. Res., Part A 2019, 107, 2123. 10.1002/jbm.a.36723 31094049

[13] M. L. Amin , D. Mawad , S. Dokos , P. Koshy , P. J. Martens , C. C. Sorrell , Mater. Sci. Eng., C 2021, 121, 111821. 10.1016/j.msec.2020.111821 33579464

[14] M. L. Amin , D. Mawad , S. Dokos , C. C. Sorrell , Biomacromolecules 2024, 25, 3131. 38554085 10.1021/acs.biomac.4c00228

[15] G. Zhai , Y. Wang , P. Han , T. Xiao , J. You , C. Guo , X. Wu , Int. J. Biol. Macromol. 2024, 281, 135779. 39419688 10.1016/j.ijbiomac.2024.135779

[16] H. Wang , S. C. Heilshorn , Adv. Mater. 2015, 27, 3717. 25989348 10.1002/adma.201501558 PMC4528979

[17] Z. Tong , L. Jin , J. M. Oliveira , R. L. Reis , Q. Zhong , Z. Mao , C. Gao , Bioact. Mater. 2021, 6, 1375. 33210030 10.1016/j.bioactmat.2020.10.029 PMC7658331

[18] S. Asim , C. Tuftee , A. T. Qureshi , R. Callaghan , M. L. Geary , M. Santra , V. Pal , I. Namli , G. H. F. Yam , I. T. Ozbolat , Adv. Funct. Mater. 2024, 35, 2407522.

[19] M. Rizwan , A. E. Baker , M. S. Shoichet , Adv. Healthcare Mater. 2021, 10, 2100234. 10.1002/adhm.202100234 33987970

[20] S. Asim , T. A. Tabish , U. Liaqat , I. T. Ozbolat , M. Rizwan , Adv. Healthcare Mater. 2023, 12, 2203148. 10.1002/adhm.202203148 PMC10330013 36802199

[21] Z. Zhang , C. He , X. Chen , Mater. Chem. Front. 2018, 2, 1765.

[22] J. Xu , Y. Liu , S.‐h. Hsu , Molecules 2019, 24, 3005. 31430954 10.3390/molecules24163005 PMC6720009

[23] Y. Han , Y. Cao , H. Lei , Gels 2022, 8, 577. 36135289 10.3390/gels8090577 PMC9498565

[24] Z. Hao , G. Liu , L. Ren , J. Liu , C. Liu , T. Yang , X. Wu , X. Zhang , L. Yang , J. Xia , ACS Appl. Mater. Interfaces 2023, 15, 19847. 37042619 10.1021/acsami.2c23323

[25] Y. Zhang , S. Chen , X. Qin , A. Guo , K. Li , L. Chen , W. Yi , Z. Deng , F. R. Tay , W. Geng , Adv. Healthcare Mater. 2024, 13, 2400318. 10.1002/adhm.202400318 38408212

[26] A. Fesharaki‐Zadeh , Int. J. Mol. Sci. 2022, 23, 13000. 36361792 10.3390/ijms232113000 PMC9657447

[27] A. Nguyen , A. B. Patel , I. P. Kioutchoukova , M. J. Diaz , B. Lucke‐Wold , Oxygen 2023, 3, 163.

[28] X. Xu , Y. Han , B. Zhang , Q. Ren , J. Ma , S. Liu , Cell Commun. Signaling 2024, 22, 132. 10.1186/s12964-024-01509-w PMC10874090 38368403

[29] P. Batista , S. A. Cunha , T. Ribeiro , S. Borges , S. Baptista‐Silva , P. Oliveira‐Silva , M. Pintado , Trends Food Sci. Technol. 2024, 143, 104300. 10.1080/10408398.2023.2232866 37417323

[30] Y.‐S. Han , J. H. Lee , S. H. Lee , Marine Drugs 2019, 17, 518.31480724

[31] A. H. Pandit , N. Mazumdar , S. Ahmad , Int. J. Biol. Macromol. 2019, 137, 853. 31284008 10.1016/j.ijbiomac.2019.07.014

[32] W. Ding , J. Zhou , Y. Zeng , Y.‐n. Wang , B. Shi , Carbohydr. Polym. 2017, 157, 1650. 27987879 10.1016/j.carbpol.2016.11.045

[33] N. Sultana , U. Edlund , C. Guria , G. Westman , Polymers 2024, 16, 381. 38337270 10.3390/polym16030381 PMC10857238

[34] X. Sun , F. Jiang , Carbohydr. Polym. 2024, 341, 122305. 38876711 10.1016/j.carbpol.2024.122305

[35] C. O. Pandeirada , M. Achterweust , H.‐G. Janssen , Y. Westphal , H. A. Schols , Carbohydr. Polym. 2022, 291, 119540. 35698370 10.1016/j.carbpol.2022.119540

[36] J. A. Duke , A. V. Paschall , J. Glushka , A. Lees , K. W. Moremen , F. Y. Avci , J. Biol. Chem. 2022, 298 . 10.1016/j.jbc.2021.101453 PMC8689215 34838818

[37] C. Mahmoudi , N. Tahraoui Douma , H. Mahmoudi , C. E. Iurciuc , M. Popa , Int. J. Mol. Sci. 2024, 25, 7839. 39063081 10.3390/ijms25147839 PMC11277554

[38] L. Wang , C. Oliveira , Q. Li , A. S. Ferreira , C. Nunes , M. A. Coimbra , R. L. Reis , A. Martins , C. Wang , T. H. Silva , Marine Drugs 2023, 21, 302. 37233496 10.3390/md21050302 PMC10221219

[39] A. Sichert , S. L. Gall , L. J. Klau , B. Laillet , H. Rogniaux , F. L. Aachmann , J.‐H. Hehemann , Glycobiology 2021, 31, 352. 32651947 10.1093/glycob/cwaa064 PMC8091464

[40] M. Alghazwi , S. Smid , S. Karpiniec , W. Zhang , Int. J. Biol. Macromol. 2019, 122, 255. 30401646 10.1016/j.ijbiomac.2018.10.168

[41] Sigma‐Aldrich, “*Porcine Gelatin*”, can be found under, https://101453//www.sigmaaldrich.com/US/en/product/sigma/g1890.

[42] K. Karakyriazis , V. Lührs , S. Stößlein , I. Grunwald , A. Hartwig , Mater. Adv. 2023, 4, 1648.

[43] S. Li , M. Pei , T. Wan , H. Yang , S. Gu , Y. Tao , X. Liu , Y. Zhou , W. Xu , P. Xiao , Carbohydr. Polym. 2020, 250, 116922. 33049836 10.1016/j.carbpol.2020.116922

[44] O. Chaudhuri , J. Cooper‐White , P. A. Janmey , D. J. Mooney , V. B. Shenoy , Nature 2020, 584, 535. 32848221 10.1038/s41586-020-2612-2 PMC7676152

[45] O. Jeon , T.‐H. Kim , E. Alsberg , Acta Biomater. 2021, 136, 88. 34563721 10.1016/j.actbio.2021.09.032 PMC8627484

[46] M. Guvendiren , J. A. Burdick , Nat. Commun. 2012, 3, 792. 22531177 10.1038/ncomms1792

[47] M. C. Lampi , M. Guvendiren , J. A. Burdick , C. A. Reinhart‐King , ACS Biomater. Sci. Eng. 2017, 3, 3007. 33418721 10.1021/acsbiomaterials.6b00633

[48] J. F. Betz , Y. Cheng , C.‐Y. Tsao , A. Zargar , H.‐C. Wu , X. Luo , G. F. Payne , W. E. Bentley , G. W. Rubloff , Lab Chip 2013, 13, 1854. 23559159 10.1039/c3lc50079a

[49] S. W. Choi , W. Guan , K. Chung , Cell 2021, 184, 4115. 34358468 10.1016/j.cell.2021.07.009 PMC8372535

[50] E. Saepudin , E. Sinurat , I. A. Suryabrata , IOP Conf. Series: Mater. Sci. Eng. 2018, 299, 012027.

[51] R. Pappalardo , M. Boffito , C. Cassino , V. Caccamo , V. Chiono , G. Ciardelli , ACS Omega 2024, 9, 45774. 39583672 10.1021/acsomega.4c03157 PMC11579714

[52] X. Hu , A. M. Jazani , J. K. Oh , Polymer 2021, 230, 124024.

[53] K. Rajamäki , T. Nordström , K. Nurmi , K. E. Åkerman , P. T. Kovanen , K. Öörni , K. K. Eklund , J. Biol. Chem. 2013, 288, 13410. 23530046 10.1074/jbc.M112.426254 PMC3650379

[54] L. Cao , T. Huang , X. Chen , W. Li , X. Yang , W. Zhang , M. Li , R. Gao , Oncol. Rep. 2021, 46, 228. 34476504 10.3892/or.2021.8179

[55] S. Hajjar , X. Zhou , Trends Immunol. 2023, 44, 807. 37714775 10.1016/j.it.2023.08.008 PMC10543622

[56] W. Feng , Z. Wang , Adv. Sci. 2023, 10, 2303326. 10.1002/advs.202303326 PMC10558674 37544909

[57] T. Sakai , T. Fujiyabu , Drug Deliv. System 2019, 34, 186.

[58] J. F. Woessner Jr , FASEB J. 1991, 5, 2145. 1850705

[59] H.‐J. Ra , W. C. Parks , Matrix Biol. 2007, 26, 587. 17669641 10.1016/j.matbio.2007.07.001 PMC2246078

[60] S. Hafeez , H. W. Ooi , F. L. Morgan , C. Mota , M. Dettin , C. Van Blitterswijk , L. Moroni , M. B. Baker , Gels 2018, 4, 85. 30674861 10.3390/gels4040085 PMC6318581

[61] S. Basu , S. Pacelli , A. Paul , Acta Biomater. 2020, 105, 159. 31972367 10.1016/j.actbio.2020.01.021

[62] F. Picchioni , H. Muljana , Gels 2018, 4, 21. 30674797 10.3390/gels4010021 PMC6318606

[63] M. Grosjean , L. Guth , S. Déjean , C. Paniagua , B. Nottelet , Adv. Mater. Interfaces 2023, 10, 2300066.

[64] S. Koo , R. Muhammad , G. S. Peh , J. S. Mehta , E. K. Yim , Acta Biomater. 2014, 10, 1975. 24456758 10.1016/j.actbio.2014.01.015

[65] Z. Yu , Q. Li , X. He , X. Wang , Y. Wen , L. Zeng , W. Yu , P. Hu , H. Chen , Eur. Polym. J. 2023, 197, 112330.

[66] J. Dimitrova‐Shumkovska , L. Krstanoski , L. Veenman , Marine Drugs 2020, 18, 242. 32380741 10.3390/md18050242 PMC7281157

[67] P. G. T. Perera , O. Bazaka , K. Bazaka , D. Appadoo , R. J. Croft , R. J. Crawford , E. P. Ivanova , J. Neurol. Neuromed. 2019, 4, 35.

[68] Y. Li , E. McGowan , S. Chen , J. Santos , H. Yin , Y. Lin , Marine Drugs 2023, 21, 128. 36827169 10.3390/md21020128 PMC9961398

[69] H. Yu , Q. Zhang , A. A. Farooqi , J. Wang , Y. Yue , L. Geng , N. Wu , Carbohydr. Polym. 2023, 324, 121555. 37985117 10.1016/j.carbpol.2023.121555

[70] H. Do , S. Pyo , E.‐H. Sohn , J. Nutr. Biochem. 2010, 21, 671. 19576750 10.1016/j.jnutbio.2009.03.013

[71] Y. Q. Cui , L. J. Zhang , T. Zhang , D. Z. Luo , Y. J. Jia , Z. X. Guo , Q. B. Zhang , X. Wang , X. M. Wang , Clin. Exp. Pharmacol. Physiol. 2010, 37, 422.19843098

[72] A. M. K. Jayasinghe , K. G. I. S. Kirindage , I. P. S. Fernando , K.‐N. Kim , J.‐Y. Oh , G. Ahn , Marine Drugs 2023, 21, 347. 37367672 10.3390/md21060347 PMC10303138

[73] G. Stifano , A. J. Affandi , A. L. Mathes , L. M. Rice , S. Nakerakanti , B. Nazari , J. Lee , R. B. Christmann , R. Lafyatis , Arthritis Res. Ther. 2014, 16, R136. 24984848 10.1186/ar4598 PMC4227089

[74] N. Parameswaran , S. Patial , Gene Expression 2010, 20, 87. 10.1615/critreveukargeneexpr.v20.i2.10 PMC3066460 21133840

[75] Q. Xue , Y. Yan , R. Zhang , H. Xiong , Int. J. Mol. Sci. 2018, 19, 3805. 30501075 10.3390/ijms19123805 PMC6320759

[76] Z.‐J. Xu , Y. Gu , C.‐Z. Wang , Y. Jin , X.‐M. Wen , J.‐C. Ma , L.‐J. Tang , Z.‐W. Mao , J. Qian , J. Lin , Oncoimmunology 2020, 9, 1683347. 32002295 10.1080/2162402X.2019.1683347 PMC6959428

[77] Y. Suzuki , M. Shirai , K. Asada , H. Yasui , M. Karayama , H. Hozumi , K. Furuhashi , N. Enomoto , T. Fujisawa , Y. Nakamura , Sci. Rep. 2018, 8, 13129. 30177769 10.1038/s41598-018-31565-5 PMC6120933

[78] Z. Li , L. Wang , Y. Ren , Y. Huang , W. Liu , Z. Lv , L. Qian , Y. Yu , Y. Xiong , Cell Death Discovery 2022, 8, 413. 36209203 10.1038/s41420-022-01200-4 PMC9547100

[79] E. Apostolova , P. Lukova , A. Baldzhieva , P. Katsarov , M. Nikolova , I. Iliev , L. Peychev , B. Trica , F. Oancea , C. Delattre , Polymers 2020, 12, 2338. 33066186 10.3390/polym12102338 PMC7602053

[80] J. Huang , J. Ren , G. Chen , Z. Li , Y. Liu , G. Wang , X. Wu , Mater. Sci. Eng., C 2018, 89, 213. 10.1016/j.msec.2018.04.009 29752091

[81] Z. Emami , M. Ehsani , M. Zandi , R. Foudazi , Carbohydr. Polym. 2018, 198, 509. 30093028 10.1016/j.carbpol.2018.06.080

[82] S. Asim , E. Hayhurst , R. Callaghan , M. Rizwan , Int. J. Biol. Macromol. 2024, 264, 130657. 38458282 10.1016/j.ijbiomac.2024.130657 PMC11003839

[83] M. Rizwan , C. Ling , C. Guo , T. Liu , J. X. Jiang , C. E. Bear , S. Ogawa , M. S. Shoichet , Adv. Healthcare Mater. 2022, 11, 2200880. 10.1002/adhm.202200880 36180392

[84] H. Park , X. Guo , J. S. Temenoff , Y. Tabata , A. I. Caplan , F. K. Kasper , A. G. Mikos , Biomacromolecules 2009, 10, 541. 19173557 10.1021/bm801197m PMC2765566

[85] B. Wiatrak , A. Kubis‐Kubiak , A. Piwowar , E. Barg , Cells 2020, 9, 958. 32295099 10.3390/cells9040958 PMC7227003

