# Supplementary material for: Imine Crosslinked, Injectable, and Self‐Healing Fucoidan Hydrogel with Immunomodulatory Properties
Source: Adv Healthc Mater. 2025 Apr 18;14(15):2405260. doi: 10.1002/adhm.202405260 (PMC12147990; doi:10.1002/adhm.202405260)
Supplement: Supplementary file 1 — Supporting Information [file ADHM-14-0-s001.docx]

**Supporting information**

**Imine Crosslinked, Injectable and Self-healing Fucoidan Based Hydrogels with Immunomodulatory Properties**

Asma Talib Qureshi^1^, Shajia Afrin^2^, Saad Asim^1^, and Muhammad Rizwan^1,3,*^

^1^Department of Biomedical Engineering, University of Texas Southwestern Medical Center, Dallas, TX, 75235

^2^Department of Biomedical Engineering, Michigan Technological University, Houghton, MI, 49931

^3^Department of Ophthalmology, University of Texas Southwestern Medical Center, Dallas, TX, 75235

*Corresponding author: Dr. Muhammad Rizwan. Contact: [muhammad.rizwan@utsouthwestern.edu](mailto:muhammad.rizwan@utsouthwestern.edu)

**Table S1**. Degree of oxidation of 3 different batches of OFu

|  | **Batch 1** | **Batch 2** | **Batch 3** | **Average ± SD** |
| --- | --- | --- | --- | --- |
| **Degree of oxidation (%)** | 33 | 33.90 | 32.76 | 33.22 ± 0.49 |


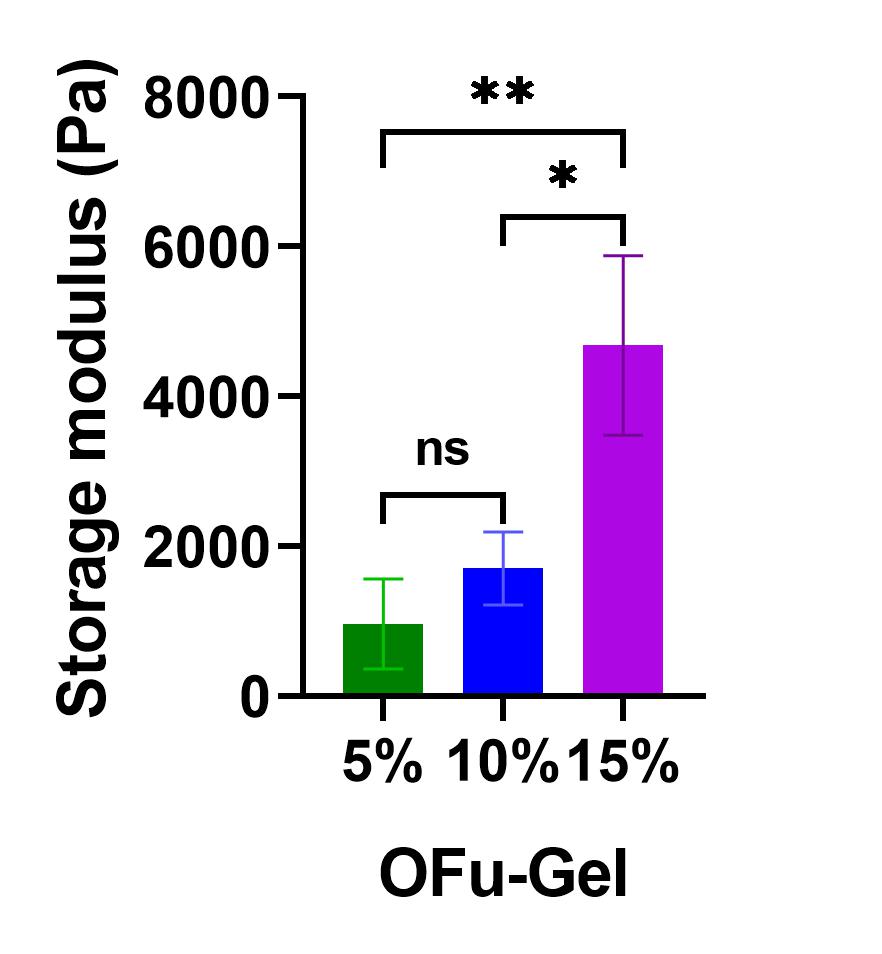


**Figure S1.** Storage moduli of 5%, 10% and 15% OFu-Gel hydrogels monitored at 1Hz frequency by applying 1% constant strain. P** < 0.01, P* < 0.05, one-way ANOVA, Tukey’s multiple comparisons test. n = 3, mean ± SD


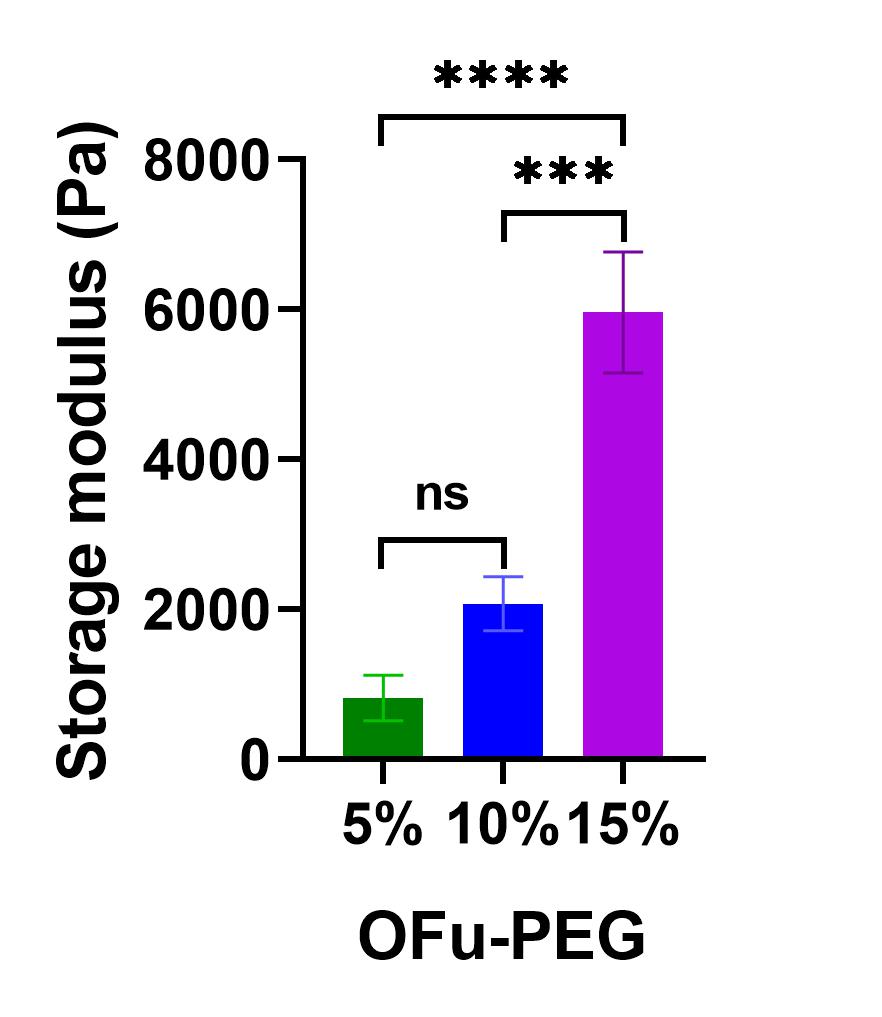


**Figure S2.** Storage moduli of 5%, 10% and 15% OFu-PEG hydrogels monitored at 1Hz frequency by applying 1% constant strain. P**** < 0.0001, P*** < 0.001, one-way ANOVA, Tukey’s multiple comparisons test. n = 3, mean ± SD


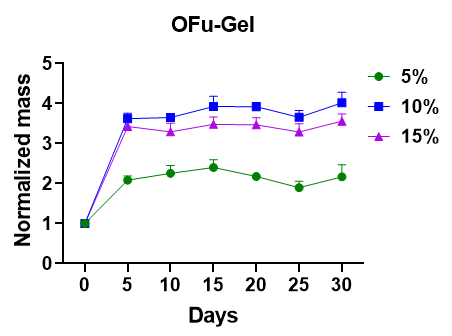


**Figure S3.** In-vitro stability analysis of 5%, 10% and 15% OFu-Gel hydrogels when incubated in PBS for a month. The mass of hydrogels measured on each day was normalized by dividing it with the weight on day 0, taken prior to incubation in any medium.


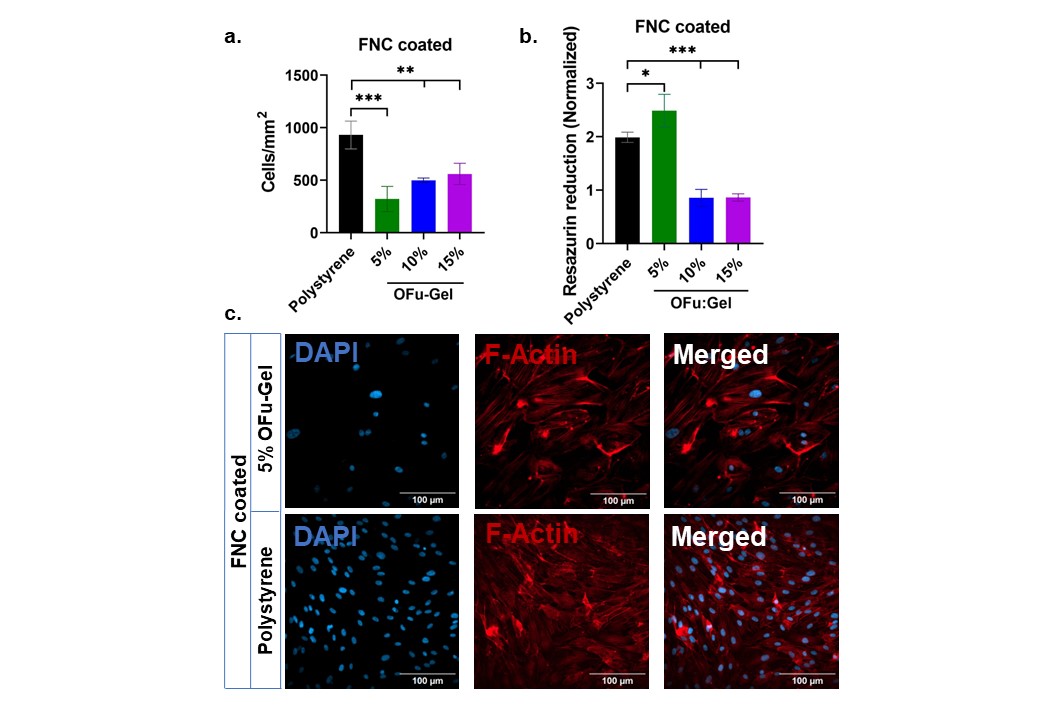


**Figure S4.** Effect of FNC coating on cell adhesion, viability and spreading of TIME-GFP cells. **(a)** TIME-GFP cell adhesion after 3 hours of initial seeding on FNC coated 5%, 10% and 15% OFu-Gel and polystyrene. **(b)** Fold change in metabolic activity of TIME-GFP cells cultured on FNC coated 5%, 10% and 15% OFu-Gel hydrogels, and polystyrene observed on day 7 and normalized to metabolic activity monitored on day 1. P*** < 0.001, P** < 0.01, P* < 0.05, one-way ANOVA, Tukey’s multiple comparisons test. **(c)** F-Actin staining showing TIME-GFP cell spreading on FNC coated 5% OFu-Gel hydrogel and polystyrene. Scale bar represents 100 µm. n = 3, mean ± SD for all


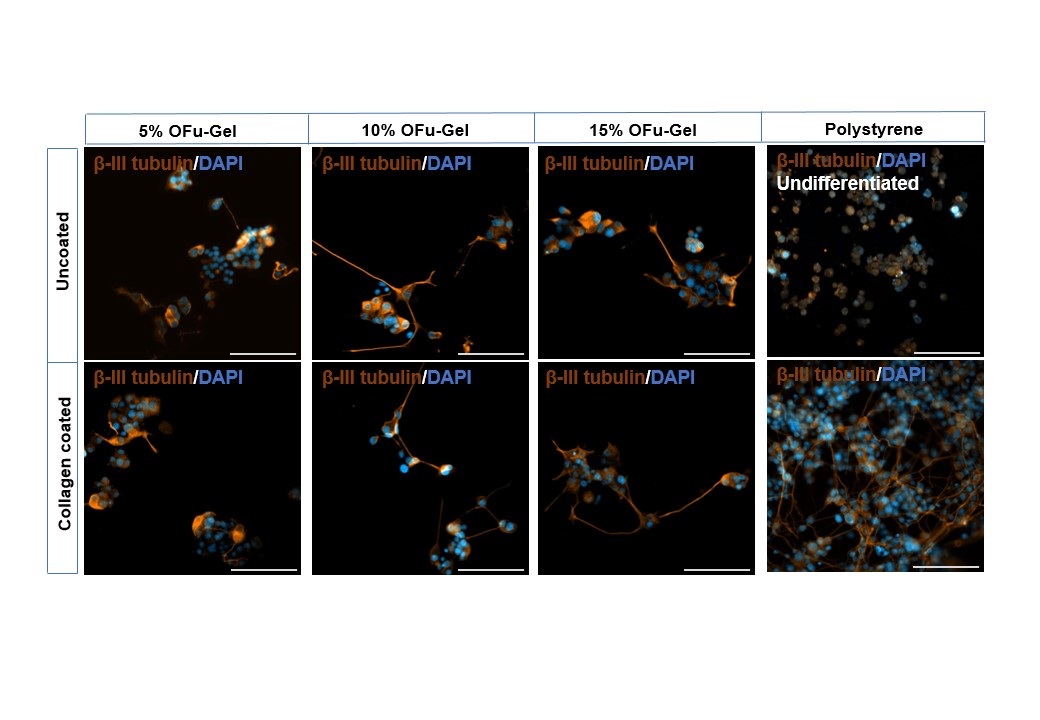


**Figure S5**. Axonal projections of differentiated PC-12 cells visualized by β-III tubulin staining of microtubular network. Scale bar, 100 µm.


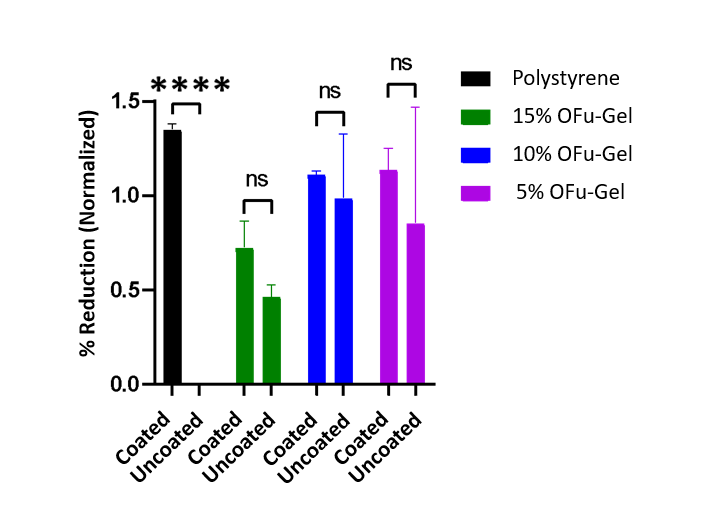


**Figure S6.** Fold change in metabolic activity of differentiated PC-12 cells observed on day 7. Data normalized to metabolic activity on day 1. Two-way ANOVA, Sidak’s multiple comparisons test, n=3, mean ± SD for all.

**Table S2.** List of primary and secondary antibodies used for IHC

|  | **Antibody** | **Catalog #** | **Dilution** |
| --- | --- | --- | --- |
| **Primary antibody** | TNF alpha Antibody | sc-52746 (Santa Cruz Biotechnology Inc) | 1:100 |
|  | NOS2/iNOS Antibody | sc-7271 (Santa Cruz Biotechnology Inc) | 1:100 |
|  | Arginase-1 (D4E3M™) XP® Rabbit mAb (Alexa Fluor® 488 Conjugate) | 66297S (cell signaling technologies) | 1:100 |
|  | CD206/MRC1 (E6T5J) XP® Rabbit mAb | 24595S (cell signaling technologies) | 1:100 |
| **Secondary antibody** | Alexa Fluor 546 F(ab') 2 fragment of goat anti-mouse lgG (H+L) | A-11018 (Invitrogen) | 1:500 |
|  | Alexa Fluor 488 F(ab') 2 fragment of goat anti-rabbit lgG (H+L) | A-11070 (Invitrogen) | 1:500 |


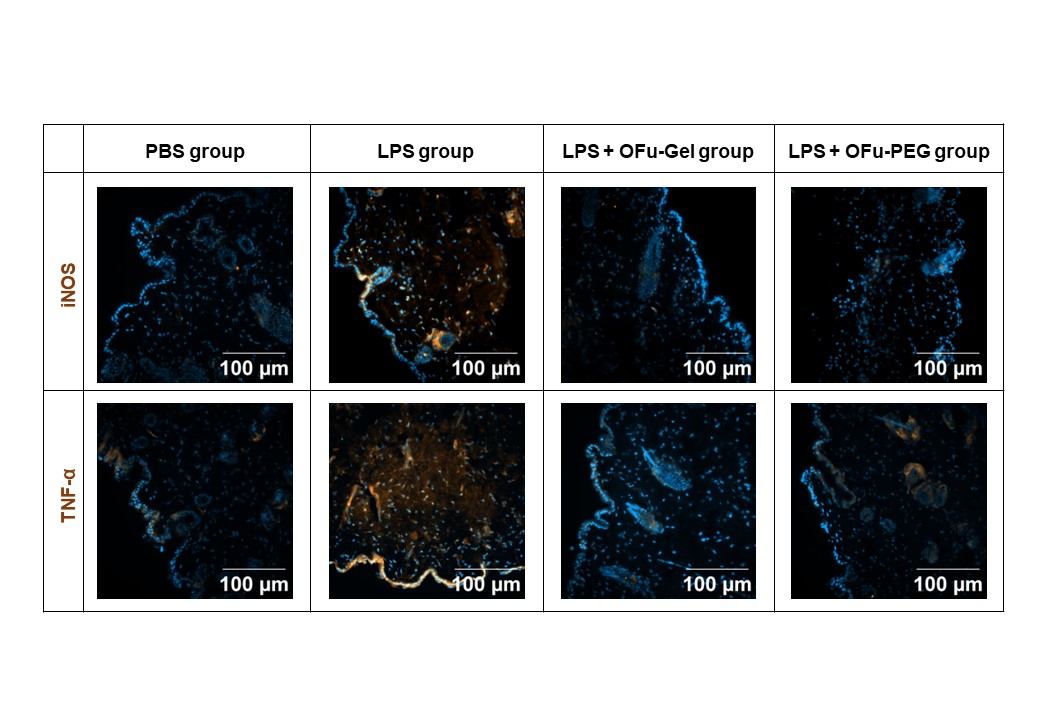


**Figure S7.** IHC showing expression of pro-inflammatory markers (iNOS and TNF-α) by skin tissue collected after 7 days of subcutaneous injection of OFu-Gel and OFu-PEG hydrogels. Scale bar represents 100 µm.


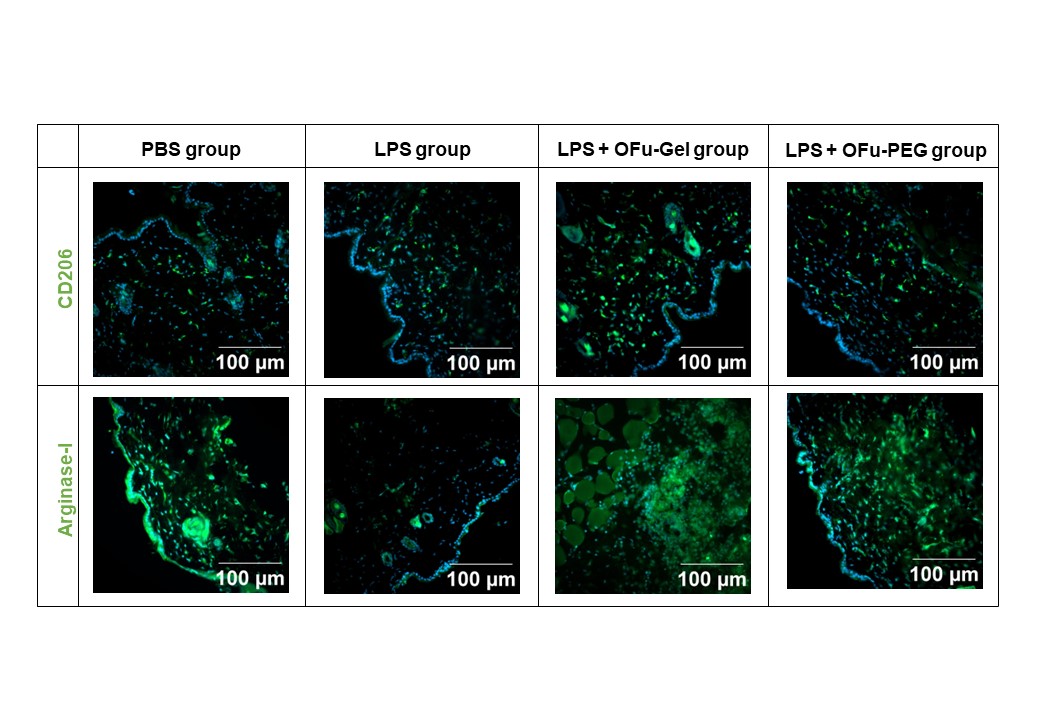


**Figure S8.** IHC showing expression of anti-inflammatory markers (CD206 and arginase-I) by skin tissue collected after 7 days of subcutaneous injection of OFu-Gel and OFu-PEG hydrogels. Scale bar represents 100 µm.
